# High-throughput detection of T-DNA insertion sites for multiple transgenes in complex genomes

**DOI:** 10.1186/s12864-022-08918-6

**Published:** 2022-10-05

**Authors:** Brianne Edwards, Eli D. Hornstein, Nathan J. Wilson, Heike Sederoff

**Affiliations:** Department of Plant and Microbial Biology, North Carolina State University, Raleigh, NC 27695 USA

**Keywords:** Transgene, Insertion site, Polyploid, T-DNA, Sequencing

## Abstract

**Background:**

Genetic engineering of crop plants has been successful in transferring traits into elite lines beyond what can be achieved with breeding techniques. Introduction of transgenes originating from other species has conferred resistance to biotic and abiotic stresses, increased efficiency, and modified developmental programs. The next challenge is now to combine multiple transgenes into elite varieties via gene stacking to combine traits. Generating stable homozygous lines with multiple transgenes requires selection of segregating generations which is time consuming and labor intensive, especially if the crop is polyploid. Insertion site effects and transgene copy number are important metrics for commercialization and trait efficiency.

**Results:**

We have developed a simple method to identify the sites of transgene insertions using T-DNA-specific primers and high-throughput sequencing that enables identification of multiple insertion sites in the T_1_ generation of any crop transformed via *Agrobacterium*. We present an example using the allohexaploid oil-seed plant *Camelina sativa* to determine insertion site location of two transgenes.

**Conclusion:**

This new methodology enables the early selection of desirable transgene location and copy number to generate homozygous lines within two generations.

**Supplementary Information:**

The online version contains supplementary material available at 10.1186/s12864-022-08918-6.

## Background

Despite great advances in crop breeding and gene editing technologies, introduction of many novel traits into elite crop lines requires transfer of genes across species barriers, generating transgenic plants. Many of these traits are focused on herbicide and insect resistance and were widely used as single gene traits [1]. Unsurprisingly, wide application of pesticides to pesticide-resistant crops has led to the evolution of resistances in weeds and insects. Weed species have evolved resistance to every herbicide class in use and new effective herbicides have not been brought to market [2, 3]. To overcome the challenges of resistance evolution in weeds and insects causing high economic losses, stacking/pyramiding of transgenes combined with integrated pest management strategies are being developed and employed [4, 5]. Gene stacking has proven especially successful in combining different resistance (R-) genes against pathogens into elite or wild lines [6, 7]. New research has identified many single gene traits that can confer abiotic and biotic resistance or increase yield, flowering time or pathogen resistance that will also be combined into elite commercial lines [4, 8]. Polygenic agronomic traits can also be improved by multiple gene transformation. Plants with stacked traits occupied nearly 41% of the global genetically modified crops area of 185 million hectares in 2016, with increasing tendency [9].

Gene stacking or pyramiding can be achieved by different approaches: co-transformation of individual transgenes or multigene cassettes or crossing of already transgenic lines. Each of these methods has different advantages and challenges, depending on the desired traits and the target crop’s genome. Most current transformations are carried out using *Agrobacterium tumefaciens*. *Agrobacterium*-mediated methods of gene transfer are by far the most expeditious and transformation can be accomplished in many crop species to generate stable transgenic lines.

Currently, integration of the transgene-containing cassette into the crop genome via *Agrobacterium*-mediated transformation is random in location and number. This often requires an extensive amount of selection and segregation following the initial transformation event to identify lines with the desired transgene number and no insertion-site effects. For trait characterization, homozygous lines (*i.e.* the T-DNA is present in both alleles at the insertion locus) are usually preferred due to the stable inheritance of the trait and known dosage effects.

The first transgenic generation (T_1_) following *Agrobacterium* transformation of a diploid plant is heterozygous for the transgene. In the simplest case, in which the trait segregates following Mendelian fashion, it is relatively easy to achieve homozygosity in the T_2_ generation with about 25% of T_2_ individuals being homozygous. The genotype of these individuals is frequently identified through segregation analysis. This simple method uses the ratio of T-DNA inheritance to understand the parental genotype [10]. In the case of gene-stacking, however, this method becomes much more laborious. With just two traits to be stacked, the chances of achieving homozygosity in the T_2_ generation for both traits drops considerably to 6.25%. With three or more T-DNA insertions, the probability decreases to 1.67%. This decreasing probability necessitates growing and analyzing hundreds of plants and often requires more generations of plants, thereby increasing the time before trait characterization can begin.

An additional layer of complexity is that the T-DNA insertion process of *Agrobacterium*-mediated transformation often leads to multiple insertions of a trait within the genome [11]. This muddles the process of segregation analysis as the trait will no longer follow typical Mendelian ratios. This propensity for multiple insertion sites can lead to troublesome false positives in selecting for homozygous lines, in which progeny assumed to be homozygous, are in fact segregating for the trait at multiple insertions. Simple analysis of a trait’s zygosity through marker segregation does not easily allow the detection of this or other issues, such as chromosomal rearrangements, translocations or T-DNA repeats [12, 13]. Insertions, deletions and complex rearrangements of both T-DNA and the host genome are in fact very common. Recent studies have revealed large translocations and epigenetic modifications even in the most commonly used *Arabidopsis* T-DNA insertion lines [14]. This phenomenon creates difficulties in finding the precise location of a T-DNA insertion, frequently only being able to identify one end of the T-DNA sequence [14, 15]. This prevalence of aberrant insertion has almost become the “rule” of T-DNA insertion, rather than the exception. These insertion phenomena present not just a challenge in zygosity determination, but also affect basic genetic understanding, are influential in transgene silencing, and may impact regulatory approval [4]. Therefore, it has become necessary for many researchers, especially in the case of gene-stacking, to characterize the T-DNA insertion locus using several different approaches. With advances in sequencing technology, this process has become easier but still presents several challenges for large-scale adoption. The continuing barriers to adoption mean that insertion site detection has yet to become a part of the workflow for transgenic experiments where it has well-documented biological impacts and obvious practical utility.

For decades genome-walking methods have been used to amplify unknown genomic sequences with varying degrees of success. Here, we utilized one of the more recent variations on this method from Kalendar et al. [16, 17] which was designed to alleviate some of the major bottlenecks that are commonly encountered in genome-walking PCR and also addresses some limitations of insertion-site finding methods. By combining this method with high-throughput sequencing, we show in an example using diploid *Arabidopsis thaliana* and allohexaploid *Camelina sativa* transgenic lines how detection of insertion sites in the T_1_ generation of two stacked transgenic camelina lines can be quickly and easily characterized. We show how this information can be used for early selection of homozygous lines with defined copy number and insertion sites in the allohexaploid *Camelina sativa.*

## Results

### Overview of experimental workflow

**Fig. 1:**
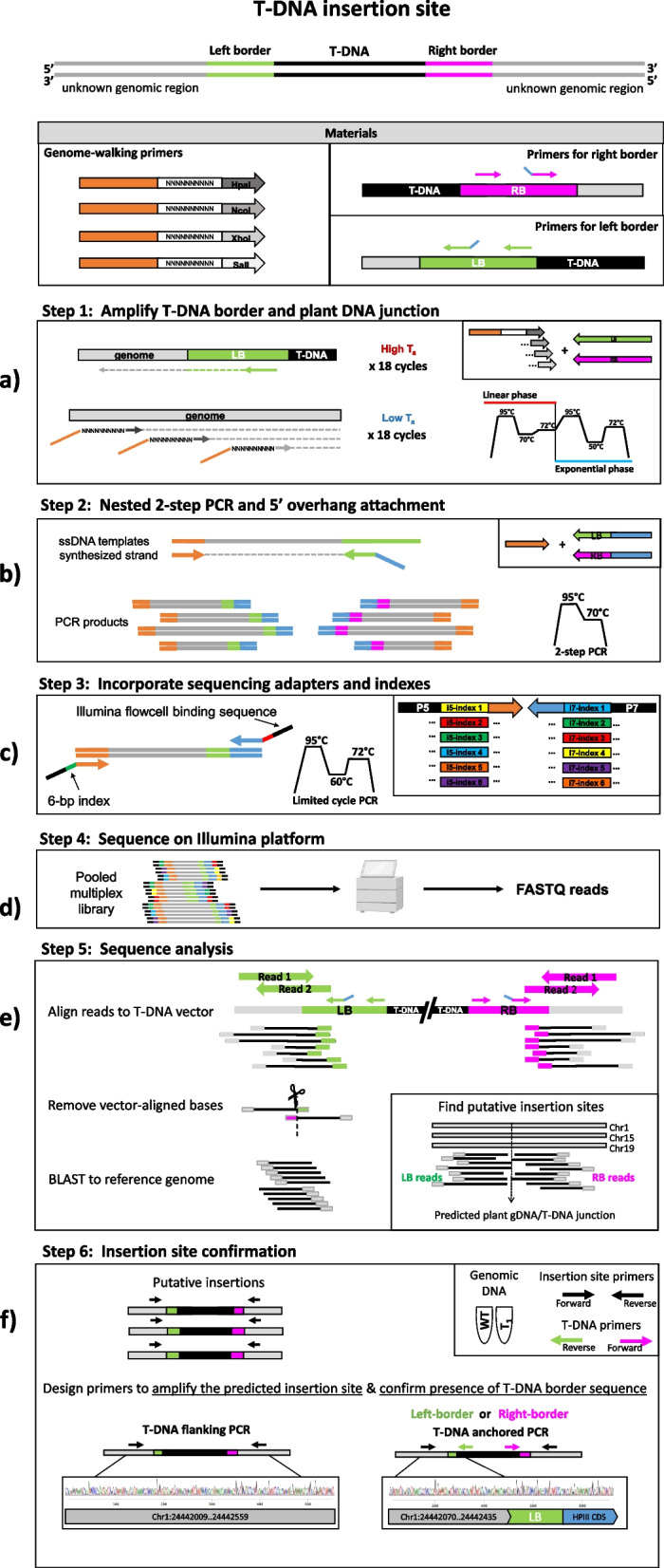
Schematic representation of the materials and overview of the method. **a** The transgene border and adjacent plant genomic sequence are amplified in a single PCR consisting of two phases: a high-temperature linear phase which favors transgene-specific primer binding and extension, and a low-temperature exponential phase to favor binding of genome walking primers to the linearly amplified transgene-containing template. **b** PCR products from step one are used in a second PCR to attach 5’ overhang and enrich for transgene-containing targets using a separate, nested transgene-specific reverse primer. with a 5’ overhang sequence for adapter attachment. **c** PCR is performed to incorporate unique barcodes and a P5 and P7 Illumina adapter. **d** Indexed PCR amplicons are pooled together into a single multiplex sequencing library and sequenced on an Illumina platform. **e** Sequences are processed as single-end reads starting with alignment of the transgene-containing read (read 2) to the transgene border / vector backbone sequence. Remaining unaligned regions are submitted as BLAST database query against the reference genome of the transformed species. Left- and right-border derived libraries are processed independently. Final BLAST results are then combined and compared to identify “hotspots” or regions of high read support across multiple libraries to obtain a list of putative insertion sites for further investigation. **f** Primers are designed to span the predicted insertion site and amplify the full genomic region in the “T-DNA flanking PCR”. A second PCR is performed with a forward or reverse insertion site-specific primer along with a transgene-specific primer in the “T-DNA anchored PCR” to confirm the presence of a T-DNA/plant DNA junction. PCR products are then sanger sequenced to verify the sequence of the plant genomic region associated with the insertion using longer read lengths

In this study, we utilize a genome-walking PCR approach and demonstrate how it can be easily adapted for high-throughput screening of multiple lines and transgenes at one time on a benchtop, short-read sequencing platform. Insertion sites identified in this sequencing can then be used to design screening primers to test for zygosity of subsequent generations at every locus and also enable segregation of irregular or silent insertions. The overall process of identification of transgene insertion sites is accessible, as it involves commonly used laboratory techniques and can be completed in 6 steps (Fig. 1):

1. Amplify the transgene border and adjacent plant genomic sequence. This is accomplished in a single PCR with two distinct phases. First, a highly specific primer anneals at a high temperature to the left- or right-border sequence of the transgene to initiate linear amplification and extension from the transgene border sequence. Second, a low-temperature annealing phase allows semi-random genome walking primers to bind to the partially enriched transgene-containing fragments amplified in phase one and amplify these exponentially (Fig. 1a).
2. Nested PCR and 5’ overhang attachment. PCR products from step one are used as templates for a second PCR to attach 5’ overhang and further enrich for transgene-containing targets using a separate, nested transgene-specific reverse primer with a 5’ overhang sequence for adapter attachment (Fig. 1b).
3. PCR to incorporate sequencing adapters and indexes. A final PCR is performed which utilizes the 5’ overhang regions on either end of the amplicon to attach unique barcodes and a P5 and P7 Illumina adapter. Individual PCR reactions can then be pooled together into a single multiplex sequencing library (Fig. 1c).
4. Sequencing on an Illumina platform. The pooled library is size selected for amplicons between 150 bp and 1.2 kb. Concentrations are measured for each size range and used for library normalization to balance representation of large and small amplicons in the final library prior to sequencing (Fig. 1d).
5. Sequence analysis. The paired-end sequencing library is processed as single-end reads, starting with alignment of the transgene-containing read (read 2) to the transgene border and vector backbone sequence, and the remaining unaligned regions are submitted as BLAST database query against the reference genome of the transformed species to identify high-identity matches from both left- and right-border derived libraries independently. Results for each line are then combined and compared to identify “hotspots” or regions of high read support across multiple libraries to obtain a list of putative insertion sites for further investigation (Fig. 1e).
6. Insertion site confirmation. Primers are designed to amplify across the predicted insertion site and each forward or reverse primer is used to independently test for the presence of a T-DNA border/plant DNA junction. Amplified products are then Sanger sequenced to verify the sequence of the plant genomic region associated with the insertion as well as the identity of the T-DNA inserted at that locus (Fig. 1f).

### Primer design

The greatest benefit of this method comes from features of the genome-walking primers which have improved the cost, optimization time, and performance of genome-walking PCR but also allows for easy customization to different templates. Walking primers contain a long degenerate region with a range of possible targets and annealing temperatures, however, when used in combination with a highly-specific primer for a known sequence such as the T-DNA left- or right- border, the thermal cycling conditions can be modified to favor the initial annealing and extension from the known region early in the PCR and avoid random amplification of non-border-containing products. Binding of the genome-walking primers can occur in a later, low-temperature phase of the PCR and instead of randomly annealing, binding is targeted to specific restriction motifs (due to the presence of a fixed 6-bp palindromic sequence at the 3’ end) which can be customized/pre-selected based on characteristics of the background genome being transformed.

## Transgene insertion site detection in diploid and polyploid species

To demonstrate the utility of detecting T-DNA insertion sites in the first generation of multi-construct lines in a cost-effective and high-throughput manner, we took advantage of an integration line of *Camelina sativa* that had been developed in our lab as a means to test our approach. This line was co-transformed with two different transgenes (PC-GW > Hyg::OCP1 and pCAMBIA > Bar::OGC) into *Camelina sativa* var Calena via *Agrobacterium* using the floral dip / vacuum infiltration method. Two different binary vectors, pCAMBIA-BAR (GenBank accession: KP795973.1) [18] and PC-GW series [19], were used for transformation. Two independent T_1_ lines (hereafter referred to as lines #1415 and #1416) were confirmed positive for both transgene coding sequences by PCR and chosen for downstream library construction. In addition to these, we included one non-transgenic wildtype *C. sativa* plant as a negative control as well as one characterized *A. thaliana* T-DNA insertion line from the GABI-Kat collection [20] to serve as a positive control.

Sequencing libraries were prepared from genomic DNA following the PCR steps outlined in Fig. 1. PCRs were conducted for the left- and right-border separately in order to increase the probability of detection for all possible insertions, regardless of any disassociation or disagreement between the two borders. In the first PCR reaction, a semi-random genome walking primer is used in conjunction with a border-specific reverse primer to amplify genomic sequence proximal to the transgene integration site (Table 1). The border-specific primer used in this first PCR is designed to bind ~ 200 bp from the expected left- and right-border junctions in order to avoid regions that are commonly reported to be truncated or deleted [21]. The amplified products from step one are then used as template in a second PCR reaction using a universal adapter forward primer along with a nested transgene-specific reverse primer containing a 5’ overhang sequence. Using an additional border-specific primer that binds closer to the expected junction site (~ 50–75 bp from the T-DNA tandem repeat regions) serves a dual purpose in this PCR by 1) enriching for transgene-containing targets in the final product and 2) reducing the total amplicon length and thereby increasing the potential to obtain full 150 bp reads that span the border-plant genome junction. Illumina sequencing adapters and indices are then attached to the PCR amplicons in a final PCR step through the use of 5’ overhang sequence complementarity.

**Table 1:** List of all primers used for library preparation

Genome-walking primers			
	Name	Sequence	Tm(°C)
	PST1	cctacacgacgctcttccgatctnnnnnnnnnngttaac	50
	PST2	cctacacgacgctcttccgatctnnnnnnnnnnccatgg	60
	PST3	cctacacgacgctcttccgatctnnnnnnnnnnctcgag	56
	PST4	cctacacgacgctcttccgatctnnnnnnnnnngtcgac	56
	P5 5'overhang	cctacacgacgctcttccgatct	69
Transgene-specific primers			
	Name	Sequence	Tm(°C)
*Camelina sativa*	LB1	agggttcctatagggtttcgctcatgtgttgagc	77
	LB2	agacgtgtgctcttccgatctgcggacgtttttaatgtactgaattaacgc	70
	RB1	tacccaacttaatcgccttgcagcacatcc	76
	RB2	agacgtgtgctcttccgatctagcctgaatggcgaatgctagagc	71
*Arabidopsis thaliana*	LB1	gatcgtgaagtttctcatctaagcccccatttgg	77
	LB2	agacgtgtgctcttccgatcttccagatcccccgaattaattcggc	74
	RB1	catcgtggaaaaagaagacgttccaaccacg	77
	RB2	agacgtgtgctcttccgatctagcctgaatggcgaatgctagagc	71
	P7 5'overhang	agacgtgtgctcttccgatct	65

Genome walking primers and transgene-specific primers with their calculated melting temperatures (Tm). Transgene-specific primer names correspond to both the target border (LB/RB) and PCR reaction (1/2) in which they are used. Melting temperatures were calculated using a primer concentration of 0.5 μM for genome-walking primers and 0.25 μM for transgene-specific primers and only for the “core” anticipated binding sequence, excluding the 5’ overhang nucleotides. Degenerate nucleotides are represented as “n”

**Fig. 2:**
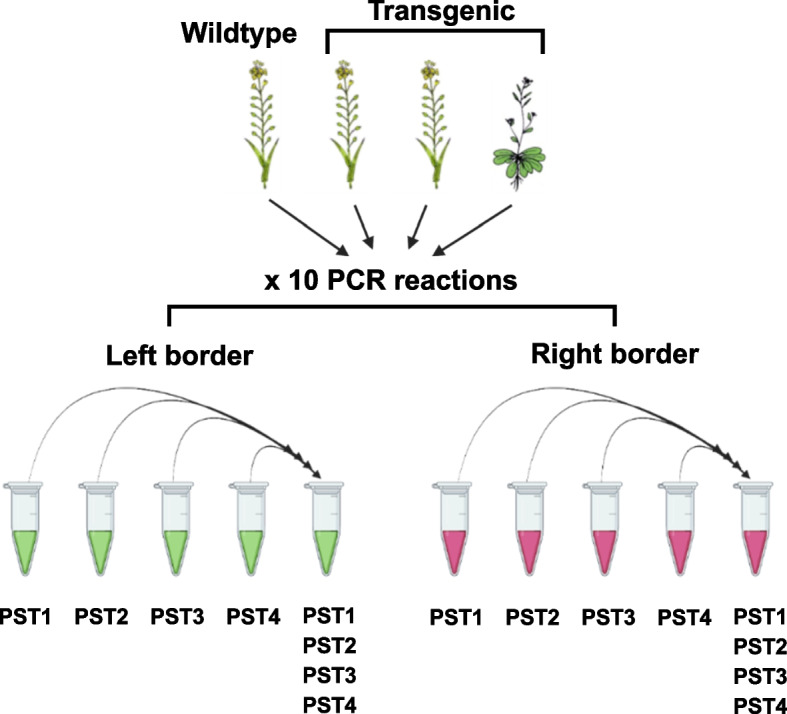
Experimental design used for insertion site detection in Arabidopsis and Camelina For each transgenic line, a total of 10 PCR reactions were performed with five corresponding to the 3’ and 5’ ends of the respective transgenes, or to the left and right border. Separate reactions were performed for all pairwise combinations of a left- or right-border and one of four genome-walking primers (denoted with a “PST” prefix) and indexed separately prior to sequencing. One representative reaction (represented here with PST primers 1-4) was pooled prior to indexing to determine if the insertion site signal was strong enough to be detected in a single reaction

The 6-bp motifs at the 3’ end of the genome-walking primers were selected based on frequency of occurrence in the *Camelina* genome (see Additional file 1 for more details). Specifically, we focused on motifs which occurred with moderate frequency across the whole genome and similar frequency between chromosomes. To compare overall efficiency and performance of the different genome-walking primers, each of the four individual reactions were indexed separately. To test whether this approach (*i.e.* separate indexing) can be scaled for even higher-throughput, we combined all four genome-walking reactions into a single “representative” library with a separate unique index. We reasoned that if one or all of the genome-walking primers was efficient at amplifying an insertion, the number of amplicons containing the insertion junction should be more abundant in relation to other products and would therefore still be detectable in a subsample of that library. The final sequencing library consisted of 5 uniquely barcoded reactions for both the left- and right-border for every transgenic and wildtype plant (Fig. 2).

Because the size of any amplified product depends on the distance from a genome-walking primer motif to a given transgene insertion site and these factors are expected to differ for each independent insertion and transgenic line, performing a strict size selection cutoff would likely result in a loss of valuable information. It is possible, however, to sequence larger amplicons (with insert sizes > 150 bp) on a short read sequencing platform in paired-end mode with the tradeoff being loss of sequence in the middle of the amplicon. We reasoned that including amplicons of various sizes would allow us to retain the maximum amount of data and in some cases, may lead to higher read support for some insertion sites if palindromic motifs for different genome-primers are within close range of one another. For this reason, dual-indexed libraries were pooled together into a single multiplex library and size selection was performed at 150 bp intervals on an agarose gel for all amplicons between 200–1200 bp. Library quantification was performed for each 150 bp amplicon interval separately and then combined in equimolar concentrations prior to sequencing.

### From sequencing results to insertion site retrieval

Overall sequencing depth per line ranged from 400–800,000 reads with approximately 48% corresponding to the left-border and 51% to right-border libraries. Raw reads were aligned to the T-DNA vector reference sequence and aligned regions of the read were trimmed following the bioinformatic methods outlined below. The remaining soft-clipped and “transgene-free” bases were retained and queried against the GenBank genomic reference sequence database for *Camelina sativa* (GCF_000633955.1) or, in the case of Arabidopsis, the TAIR10.1 reference genome (GCF_000001735.4) using the blastn command in BLAST + with the search parameters described in Methods.

The number of returned BLAST hits was greatest for the transgenic lines (between 11,000 and 57,000 hits) and only a few results were obtained for wildtype Camelina (94 total hits) (Table 2). Reads from right-border libraries produced a higher number of BLAST hits overall, however this trend was largely driven by line #1416. For the other lines, the majority of BLAST results were obtained from left-border libraries. These results are in line with the expectation that successful amplification relies on insertion-site specific characteristics which differ across transgenic lines due to the random nature of T-DNA insertion.

**Table 2:** Summary of BLAST results obtained for each line and PCR library

			Number of BLAST hits per PCR library					
Line	Total input reads	Border	PST1	PST2	PST3	PST4	Pooled	Total hits
Line #1415	1,187,867	LB	7545	262	35	139	3158	11,139
		RB	35	16	54	0	9	114
Line #1416	1,593,516	LB	5577	6578	753	57	2426	15,391
		RB	33,208	395	98	600	7327	41,628
GK-269g12	625,724	LB	0	0	11	16,923	10,391	27,325
		RB	17	20	24	32	18	111
WT	189,053	LB	10	0	64	0	7	81
		RB	0	4	9	0	0	13

The total number of reads reflects the number of informative reads remaining for each line after mapping to the T-DNA vector, removing T-DNA matching bases, and applying read length filtering. BLAST hits represent the number of reads passing the query search parameters and resulted in a corresponding match to the plant genome. Number of hits are shown for each sequencing library separately and total hits summarized across all libraries for the left- and right-border

Differences were also observed between libraries generated with different genome-walking primers (Table 2). For both transgenic Camelina lines more than 50% of all BLAST hits were obtained from a single library (primer PST1) and the same was true for Arabidopsis (primer PST4). Although results for the Camelina wildtype were minimal, 78% of all hits were obtained from the same library (primer PST3) indicating some potential for mispriming in the genome when this primer motif is used in combination with a border-specific primer sequence which has no clear target or binding site with perfect complementarity. In all cases, however, results from the pooled library were the next largest contributor to the overall BLAST hits obtained for each line which supports the notion that it is representative of the four genome-walking libraries.

Putative insertion sites were selected from the database of BLAST hits in a stepwise manner based on a hierarchical list of criteria prioritized by the strength of evidence. First, we sorted the list of BLAST hits by chromosome and position to identify regions which were detected in both left- and right-border libraries (within 1 kb). In cases where the first criterion was not satisfied, we narrowed our search to regions with the highest amount of read support regardless of border. Sites identified on the basis of read support were then weighted according to read- and library-based characteristics. Sites were ranked higher when 1) hits were detected in more than one PCR library, 2) BLAST query statistics had e-values lower than 10^–30^ or greater than 99% identity, 3) at least one read from the BLAST query exceeded 60 bp in length. Lastly, we compared the list of candidate sites for each line to every other line to ensure that sites were unique.

Results for the diploid Arabidopsis T-DNA insertion line were unambiguous. Both left- and right-border libraries contained BLAST hits corresponding to the same region on chromosome 1 within the coding region of AT1G47960, which is in line with the T-DNA flanking sequence predicted in the GABI-Kat SimpleSearch database [20]. Sequences supporting the insertion were derived from both soft-clipped and unmapped reads from read 1 of the pair. Soft-clipped regions for read 2 were shorter by comparison (~ 40–50 bp) and resulted in mostly spurious BLAST matches that were filtered in the site selection process described above.

More putative insertion sites were identified in the BLAST results for Camelina transgenic lines. In addition to having multiple T-DNAs and therefore a greater number of insertions to detect overall, the allohexaploid genome structure resulted in single reads producing BLAST hits in triplets due to high sequence similarity among homeologous genes. The BLAST query resulted in matches to 84 unique genomic regions for line #1415 and 265 for line #1416. From the list of candidates, we identified regions with the greatest amount of read support and those which met at least one of the weighting criteria were selected for further verification. For both lines, the list of candidate insertion sites included regions which were equally plausible candidates according to our selection criteria but corresponded to the same region on three different homeologous chromosomes. To narrow our list of candidates further, we performed a side-by-side comparison of read query statistics (length & percent identity in particular) in order to identify which of the three homeologs was most likely to contain the insertion. For cases in which the results were nearly identical and a single best match could not be clearly defined, all three homeologs were kept as valid candidates. As a result of the selection process, BLAST results were narrowed down to two putative insertion sites for line #1415 and three for line #1416. For three of the sites, BLAST hits to homeologous chromosomes were too similar to one another to make a clear assignment or argument for which one represented the “best” candidate above the others. For example, one of the putative sites found in line #1415 was supported by 7,228 reads matched to chromosome 5 in the BLAST results, however, 3692 of these reads matched equally well to a region on chromosome 16 and a smaller proportion of these to chromosome 7 as well. The same was true of two putative sites found in line #1416 and followed a similar pattern with the majority of reads matching to one of the three homeologs and a large proportion of these matching to the other homeologs and nearly identical in terms of the BLAST query statistics.

In addition to these, two other putative insertion sites were identified which also shared high sequence similarity with at least one other location in the genome, however, similarities were due to regions being paralogous to one another and rather than being conserved regions across three different subgenomes. For these reasons, each of the candidate sites identified are referred to generically as “site 1, 2, etc.” as each putative site represents a group of two to three chromosomal positions until the precise location is determined. In the process of developing our criteria for selection of best candidate insertions, these and other putative sites were selected and investigated in the same manner as outlined below. Those which were confirmed negative were then used to inform and refine the hierarchy of guidelines and/or the framework during the candidate selection process. The full list of candidate sites and corresponding genomic regions are available in Additional file 2.

### PCR validation of insertion sites

**Fig. 3:**
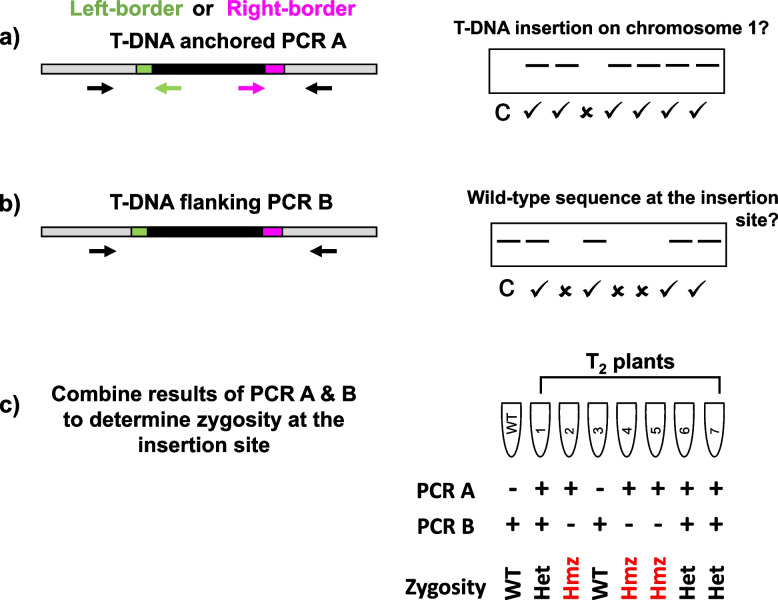
Schematic of PCR method used to determine zygosity of T_2_ generation plants Primers designed for insertion site verification of T_1_ generation plants are used to evaluate segregation of the transgene at the insertion site. The diagnostic consists of two PCRs: **a** the T-DNA anchored PCR, abbreviated as “PCR A”, utilizes one primer which binds to the plant native DNA sequence at the insertion site along with one transgene-specific primer which binds to a specific coding region within the T-DNA on either the left- or right-border to first confirm that the transgene is present and hasn’t fully segregated from the original insertion site, and **b** the T-DNA flanking PCR, abbreviated as “PCR B”, which utilizes only insertion site-specific primers to amplify the full genomic region and confirm the presence or absence of a wildtype sister chromatid. **c** Zygosity is determined by the combination of the results obtained for A/B, wherein wildtype and null segregants can be identified as -/ + heterozygous as + / + and homozygous as ±

To verify insertion sites, two PCR reactions are performed for each putative insertion site. All reactions are performed with gDNA from the transgenic T_1_ plant harboring the putative insertion and at least one wild type plant as control (Fig. 1f). For the first PCR (hereafter the “T-DNA anchored PCR”), one plant genomic (forward) primer is used in conjunction with either a left-border or right-border transgene-specific (reverse) primer to confirm the presence of a plant gDNA/T-DNA junction (Fig. 3a). In cases where putative insertions were obtained from both left- and right-border libraries, two PCRs are performed to amplify from opposite ends of the insertion to independently confirm that both flanking genomic sequences match to the predicted location. For putative sites that were obtained from only one of the libraries, it is useful to start with this end first before proceeding to confirm the other. In all cases, the orientation of mapped reads can be used to inform the pairing of transgene and plant genomic primers (see “Methods” for details). In the second PCR (the “T-DNA flanking PCR”), both plant genomic primers are used to amplify the region spanning the insertion however, due to the large size of most T-DNA constructs, the amplified product will contain only the intact wildtype sequence or the sister chromatid without the insertion (Fig. 3b). Reactions showing positive amplification for the transgenic line in the first PCR and no amplification in the wild type are then verified via sanger sequencing to confirm the presence of a T-DNA junction along with the amplified product from the second PCR to confirm primer specificity.

Using the regions identified in the BLAST hit selection process, primers were designed for each of the putative insertion sites in transgenic *Camelina* lines. Primers were designed to bind 250-500 bp on either side of the insertion site in order to meet the length requirements for sanger sequencing. PrimerBLAST was used to ensure that individual primers, both alone and in pairs, were not predicted to amplify unintended targets in the plant genome. In cases where the sequence surrounding the putative insertion site contained regions with high sequence similarity to other homeologs, additional 3’ mismatches were included to improve specificity and prevent off-target amplification if possible. Homeologous regions were nearly identical with the exception of minor SNPs, therefore primers were designed to amplify all regions non-specifically. Because the T-DNA insertion site and flanking sequence for our positive control have been validated and published in the GABI-Kat collection database [20], no additional PCR verification or sequencing was performed for the *Arabidopsis* line.

A separate set of transgene-specific primers was designed specifically for the purpose of insertion site verification in Camelina. Primers were designed to bind within the coding sequence of the BAR selectable marker for the OGC construct and within the OCP1 transgene sequence for the OCP1 construct. Careful consideration was made during design to ensure that 1) primers were unique to each transgene (~ 1 kb upstream from left- and right-border primers used for library construction), 2) the melting temperatures of all primer sequences were compatible with the genome-specific primers to be used for insertion site verification, and 3) based on Primer-BLAST screening, all transgene-specific & plant genomic primers (both alone and paired) were not predicted to amplify unintended targets in either the transgene constructs or the genome. To expedite the screening process, we also designed an additional non-specific primer to bind within the 35S promoter common to both transgenes. The full list of both transgene- and genome-specific primers used for verification are available in Supplementary Information (Additional file 2).

For each putative site, we sorted the pool of BLAST hits by subject strand (in this case, “subject” refers to our plant reference genome) to find which of the paired reads were associated with either the plus or minus strand. Because the transgene-containing end of the amplicon is closest to the P7 Illumina flow cell binding sequence which binds during read 2 synthesis, soft-clipped sequences derived from read 2 of each pair are expected to be oriented outward, or away from the transgene into the plant genomic flanking sequence, whereas soft-clipped sequences from read 1 would be expected to match to the opposite strand with sequences being oriented towards the transgene. For this reason, the strandedness of BLAST hits and whether or not the sequence was derived from read 1 or read 2 can provide valuable information that can be used to inform which of the plant genome-specific primers should be paired with the left- and right-border primers. Additionally, in cases where both left- and right-border results are obtained for a given insertion site, the configuration of the T-DNA at the integration site can be inferred in the same manner.

To test for the presence of a T-DNA/plant DNA junction at putative sites 1 and 2 in line #1415, we performed a T-DNA anchored PCR for each insertion site using a plant-genome specific forward primer which binds upstream of the predicted insertion along with a transgene-specific reverse primer adjacent to the left-border. For the initial screening process, we used a reverse primer which did not discriminate between the two different transgenes. Because putative insertion sites for line #1415 were identified based on reads from left-border libraries, PCRs targeting the region adjacent to the left-border were performed first. Aside from slight differences in the annealing temperature for individual sets of primers, the same standard thermal cycling conditions were used for all T-DNA anchored and T-DNA flanking PCRs (see Methods for details).

The same PCRs, using a plant genome forward and transgene-specific reverse primer, were performed for line #1416 to test for a left-border T-DNA junction at sites 1 and 2. Based on the strand and orientation of the original sequencing reads relative to the plant genome, we inferred that the T-DNA insertion at site 3 was in the RB-LB orientation. For example, in the right-border library, sequences derived from read 1 were matched to the + strand of *C. sativa* and read 2 sequences to the—strand, whereas in the left-border library, sequences from read 1 matched to the—strand of *C. sativa* and read 2 to the + strand and were positioned upstream relative to right-border read coordinates. Therefore, T-DNA anchored PCRs to amplify the left-border junction at site 3 specifically used a downstream genome-specific reverse primer in combination with the same transgene-specific primer used in the above PCRs (however, now it is used in the context of a forward primer rather than reverse).

T-DNA flanking PCRs were performed in tandem to verify the integrity of each primer pair and to ensure that the amplified products were specific to the intended genomic region in both wildtype and transgenic samples. Because T-DNA flanking PCRs are carried out in the first generation using genomic DNA from a heterozygous T_1_ plant, amplification is expected for both wildtype and transgenic samples. Conversely, amplification in T-DNA anchored PCRs is only expected to occur in the presence of a T-DNA border and positive amplification in the wildtype would indicate a false positive, mispriming, or possibly a genomic region with sequence characteristics much like the T-DNA border and backbone (AT-rich and repeat-rich) therefore more susceptible to mispriming.

For all T-DNA anchored PCRs, we observed positive amplification when PCRs were performed on T_1_ transgenic lines and wildtype reactions were negative, which supports the notion that a T-DNA border is associated with one of the genomic regions identified in the sequencing. To identify which of the three targets is implicated in this specific observation and responsible for amplification, all positive PCR products from transgenic samples in the T-DNA anchored PCR screening as well as the T-DNA flanking PCR products from both transgenic and wildtype samples were sanger sequenced for confirmation and subjected to further analysis.

From the sequencing results of T-DNA anchored PCRs, we were able to correctly identify which of the two transgenes were inserted at each insertion site. Sites that were successfully amplified with the transgene-specific primer contained a T-DNA junction which could be aligned to the vector and the coding sequence of the inserted transgene. Therefore, sites corresponding to an OCP1 insertion could be effectively distinguished from those containing an OGC insertion. The sequence immediately adjacent to the (non-native) inserted transgene was equally informative in that it aligned to a specific chromosome and as such we were able to resolve which of the three homeologous chromosomes or paralogous regions contained the insertion. Because primers were designed to amplify more than one target nonspecifically, Sanger sequencing from the T-DNA flanking PCR often resulted in a mixture of different products as evidenced by multiple peaks in the sequencing traces in regions of low conservation between the different templates. The sequencing traces from the T-DNA anchored PCR on the other hand did not show this same pattern and instead showed a clear signal, with high quality peaks in the electropherogram, indicating that only one of the targets was able to be amplified with the transgene-specific primer. As a result, we determined that the two insertions detected in line #1415 represented an OGC transgene insertion into a B3 domain-containing protein coding gene on chromosome 5 (site 1) and an OCP1 transgene insertion into a carnosine N-methyltransferase-like protein coding gene on chromosome 7 (site 2). For line #1416, all three sites were determined to be an OCP1 transgene insertion, two of which were located in noncoding regions on chromosomes 8 (site 1), 15 (site 2), and one within a ribosome biogenesis protein BOP1 pseudogene on chromosome 18 (site 3).

Using the sequence from the T-DNA anchored results, we designed a new primer set for the T-DNA flanking PCR to target each of the insertion sites specifically. Site-specific primer sequences are available in Additional file 2 along with the non-specific primer sequences used prior to resolving the location of the insertion. Primers were tested and amplified products were verified via Sanger sequencing to ensure that only single targets were amplified prior to being used in downstream insertion site detection PCRs. To further confirm the genomic context and configuration of the T-DNA insertion at each site, an additional T-DNA anchored PCR was performed to amplify the right-border junction with the newly designed insertion-site specific primers and to verify the sequence of the right border region of the transgene and adjacent plant genomic sequence. Results of right-border PCRs were in agreement with the left-border for three of the insertions, specifically those on chromosome 5 of line #1415 and chromosomes 15 and 18 of line #1416 (Table 3). For two of the insertions we were not able to successfully amplify from the right-border end of the transgene, however. This is consistent with a number of other studies which have reported difficulties with isolating the right-border flanking sequence in particular.

**Table 3:** Summary of confirmed insertion sites for each transgenic line

				Library support for insertion			Genomic context			T_2_ zygosity
Line	Chromosome	Location	Transgene	Border	PCR library	Total # of reads	Gene	Description	Protein	Het:Hmz:WT
Line #1415	Chr 5	9,760,996	OGC	LB	all	7228	LOC104789082	B3 domain-containing protein At2g33720-like	Plant-specific B3-DNA binding domain	5:3:0
	Chr 7	4,217,439	OCP1	LB	all	37	LOC104704287	mRNA-carnosine N-methyltransferase-like	Carnosine N-methyltransferase-like	8:0:0
Line #1416	Chr 8	24,442,089	OCP1	LB	1,3,4,5	279	none	non-coding region	none	19:0:0
	Chr 15	982,591	OCP1	LB & RB	1,2,4,5	2281	none	non-coding region	none	8:6:5
	Chr 18	7,461,224	OCP1	LB & RB	all	9236	LOC104763210	Ribosome biogenesis protein BOP1 homolog	Pseudogene	12:7:0
GK-269g12	Chr 1	17,683,348	NPTII	LB & RB	all	27,436	AT1G47960	Cell wall / vacuolar inhibitor of fructosidase 1	Cell-wall inhibitor of beta fructosidase and similar proteins	NA

The genomic location of the insertion and the transgene identified are presented on the left. Library support columns indicate which of the PCR libraries contributed to detection of the insertion site and the overall total number of reads obtained for each site. Genomic context columns provide information on the gene at the location of the insertion which may be altered by the introduction of T-DNA. Segregation of the transgene in the T_2_ generation is reported in the last column

### Determining zygosity of T_2_ generation plants

Finally, with the precise location of the inserted transgene determined and with primers specific to the transgene insertion site, PCRs can be applied to segregants of those lines to assess zygosity at every locus. The information provided by the two PCRs described above can be compared to a wildtype or separate transgenic line to screen lines for the presence of a transgene at a single locus or to find lines with specific combinations of loci that may be more desirable than others. The general process is the same as that used for the insertion site confirmation process, but unlike in the T_1_ heterozygous generation, the results of the T-DNA flanking PCR are expected to follow a mendelian ratio of positives and negatives depending on zygosity. Due to the large size of most T-DNA constructs and the limitations set by the extension time in this PCR, a homozygous individual with an insertion on both sister chromatids is no longer able to produce a product of the same size as the intact native genomic sequence. By comparing the result of both PCRs, the genotype of each individual can be determined (Fig. 3c). For example, a scenario in which the T-DNA anchored PCR is positive but negative in the T-DNA flanking PCR would follow the expectation of a homozygous individual whereas a positive for both PCRs would indicate a heterozygous individual.

To demonstrate this concept, both T-DNA flanking and T-DNA anchored PCRs were performed on the segregating progeny of lines #1415 and #1416 to determine zygosity of T_2_ generation plants for the insertions detected and confirmed in the T_1_ generation. As with the T_1_ plants, T_2_ seedlings were grown on antibiotic selection media and resistant plants were PCR confirmed for both OGC and OCP1 transgenes to ensure that only transgene positive plants were used for zygosity screening. It is important to note that prior screening on selection media is not a prerequisite for the method, however, doing so can be useful for identifying null segregants or individuals which are in fact transgenic but no longer carry a transgene at the particular locus being tested. From the selected transgene-positive T_2_ population, we obtained 8 individuals from line #1415 and 19 individuals from line #1416 to be used for downstream zygosity screening.

Based on the PCR results, we observed normal mendelian segregation for three of the five transgenes. For line #1415, the left-border junction was successfully amplified from all plants in the T-DNA anchored PCR using primers for the OGC insertion on chromosome 5 while no amplification was observed from the wildtype. Among these, five plants were positive for the native genomic sequence in the T-DNA flanking PCR including the wildtype plant indicating that, just like the T_1_ parent, these plants were heterozygous for the insertion at this locus. Three others did not show any amplification in the T-DNA flanking PCR, indicating that the native locus at the insertion site was effectively disrupted by the transgene on both sister chromatids and therefore, plants are homozygous for the OGC insertion on chromosome 5 (Additional file 4, Figure S5).

A similar pattern was observed for line #1416, where all plants were positive for the OCP1 insertion on chromosome 18 based on the T-DNA anchored PCR. In the PCR to assess zygosity, however, we observed an unexpected lack of segregation at this locus and all T_2_ progeny appeared to be heterozygous for the insertion (Table 4). To investigate this unlikely circumstance further, both the T-DNA anchored and T-DNA flanking PCR products were sanger sequenced to verify the T-DNA and genomic sequences obtained for every T_2_ individual. All left-border anchored T-DNA sequences were indeed positive for the OCP1 transgene and the adjacent plant genomic sequence was matched to the predicted genomic position on chromosome 18. However, when those adjacent sequences were aligned to the T-DNA flanking PCR result, we observed 100% alignment for only 12 samples and poor alignment for 7 samples with consistent mismatches at the same position along the sequence, indicating that the primers used in the T-DNA flanking PCR had amplified the next best target on a homeologous chromosome which was also the same length as the intended product and therefore not able to be distinguished on the basis of size via gel electrophoresis. With this knowledge, we designed an alternative set of primers to target chromosome 18 with even greater specificity than before by increasing the number of 3’ mismatches and expanding the range of the genomic interval to ensure greater differentiation between the three homeologs. Ultimately this strategy proved to be effective and upon repeating the T-DNA flanking PCR, we observed normal segregation among the T_2_ plants with positive amplification for 12 heterozygous individuals and no amplification for the 7 samples which had previously shown amplification due to mispriming, indicating that these were in fact homozygous for the insertion.

**Table 4:** Zygosity screening for T_2_ plants of line #1416 for the OCP1 insertion on chromosome 15

	Chr15:982,591				Chr18:7,461,224				Chr8:24,442,089			
**T**_**2**_** Plant**	**PCR A/B**	**Het**	**Hmz**	**WT**	**PCR A/B**	**Het**	**Hmz**	**WT**	**PCR A/B**	**Het**	**Hmz**	**WT**
1	+ / +	X			+ / +	X			+ / +	X		
2	+ / -		X		+ / -		X		+ / +	X		
3	- / +			X	+ / -		X		+ / +	X		
4	+ / +	X			+ / +	X			+ / +	X		
5	﻿+ / -		X		+ / +	X			+ / +	X		
6	- / +			X	+ / +	X			+ / +	X		
7	- / +			X	+ / -		X		+ / +	X		
8	﻿+ / -		X		+ / +	X			+ / +	X		
9	+ / +	X			+ / -		X		+ / +	X		
10	+ / -		X		+ / +	X			+ / +	X		
11	+ / +	X			+ / -		X		+ / +	X		
12	+ / +	X			+ / +	X			+ / +	X		
13	- / +			X	+ / +	X			+ / +	X		
14	- / +			X	+ / +	X			+ / +	X		
15	+ / -		X		+ / +	X			+ / +	X		
16	+ / +	X			+ / +	X			+ / +	X		
17	+ / +	X			+ / -		X		+ / +	X		
18	+ / -		X		+ / -		X		+ / +	X		
19	+ / +	X			+ / +	X			+ / +	X		
**Total trangene positive:**	**14**				**19**				**19**			
		**Het**	**Hmz**	**WT**		**Het**	**Hmz**	**WT**		**Het**	**Hmz**	**WT**
**Segregation ratio:**		**8**	**6**	**5**		**12**	**7**	**0**		**19**	**0**	**0**
		**57.14%**	**42.86%**	**35.71%**		**63.16%**	**36.84%**	**0.00%**		**100.00%**	**0.00%**	**0.00%**

Results of PCRs A and B are presented for each of the T_2_ individuals on the left. Columns on the right indicate the zygosity categorization for each T_2_ individual (denoted as “X”) evaluated on the basis of the combined result in column A/B. Segregation ratios reflect the total number of each category.

**Fig. 4:**
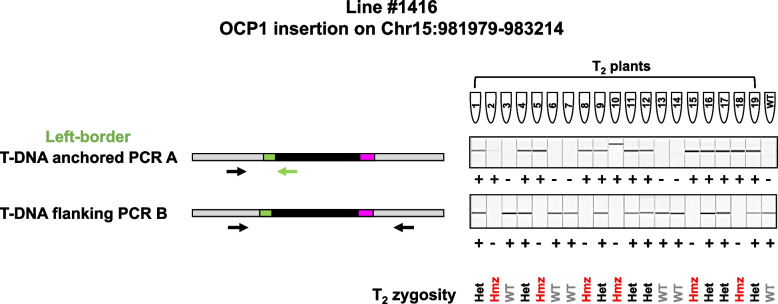
Summary of PCR results to determine zygosity of T_2_ progeny of line #1416 PCR products obtained for PCRs A and B and visualized using a capillary gel electrophoresis instrument are presented on the right. Lanes correspond to the T_2_ individuals represented in PCR tubes #1-19 and WT and are aligned in the same order for both PCRs. Full length gel electrophoresis results are provided in Additional file 4, Figures S8 and S9.

Another OCP1 insertion in line #1416 showed normal segregation, however, not all plants were positive for the insertion, indicating loss of the transgene at this locus. The OCP1 insertion on chromosome 15 segregated in a mendelian manner, eight of which were positive for the native genomic sequence and heterozygous, six plants which showed no amplification for the native allele and were therefore transgenic-homozygous, and five plants which were lacking the transgene but showed positive amplification of the native sequence (Fig. 4). Although interesting, this finding is not unexpected but instead illustrates the independent nature of transgene segregation in a multi-copy line. Since three copies of OCP1 were detected in the T_1_ generation for line #1416, each copy would be expected to segregate independently of the others (if they are on different chromosomes) and so long as one copy is present to confer antibiotic resistance, the loss of one copy can occur through segregation (Table 4). It should also be noted that without knowledge of the location of each insertion site, this scenario would most likely go undetected without extensive screening of many seedlings on antibiotic selection and counting segregation ratios and even then, the genotype does not always match the expectation based on the phenotype [22].

In both transgenic lines we observed one OCP1 insertion that did not follow the expected pattern of segregation. Amplification and Sanger sequencing of a fragment containing 900 bp of T-DNA and 550 bp of genomic DNA confirmed that the left-border region of the T-DNA was clearly integrated at the predicted genomic locations. However, PCR genotyping at these sites showed that all T_2_ progenies were heterozygous for each insertion, rather than the expected ratio of 2 heterozygotes:1 homozygote. Based on our previous observation of genotype-dependent amplification of alternative targets for the insertion on chromosome 18 in line #1416, we sequenced the PCR products of the T_2_ plants to determine whether these results could be explained by preferential binding to a homologous region or mispriming in homozygous individuals due to the absence of an amplifiable genomic target (that was not disrupted by a large T-DNA insertion). Unlike the line #1416 chromosome 18 insertion, however, in all cases the sequences obtained from both PCRs were specific to the intended target indicating that mispriming did not interfere with the PCR result and the lack of segregation at these insertion sites may be due to other reasons (Additional file 4, Figures S6 and S7). Interestingly, these two insertion sites also showed irregularities in the molecular detection method: they are the only two insertions for which the right-border-anchored PCR did not work. Many explanations are possible for the unexpected segregation and its correlation to difficulty in locating the right border. The insertions–encompassing an unknown extent of the genome towards the right border–could cause deletions that are homozygous-lethal. They may also represent more serious genomic rearrangements that disrupt chromosomal segregation itself. Although it’s possible to use the information about the left border as a starting place to hunt down the underlying explanation for the irregularities associated with these insertion sites, it would be more consistent with most transgenic applications to use the existing information and ability to PCR-screen for the left border of this insertion to simply segregate these suspect insertions out of the line altogether. Taken together, our findings for these insertions highlight some of the ways in which random T-DNA insertions may lead to outcomes that are less predictable than expected, and the utility of early detection for such cases.

## Discussion

Insertion site detection has been a topic of interest for many years, however, the majority of published methods require specialized materials, reagents, or an extensive amount of troubleshooting and are therefore only feasible for a narrow range of end-users. In addition to these barriers, it is unclear which method is more applicable as each one comes with its own set of advantages and disadvantages. For example, early methods such as TAIL-PCR which involved the use of degenerate primers and a series of amplification steps with nested transgene-specific primers were promising but tedious to implement since the success rate was highly dependent on intricacies in the thermal cycling regime and handling of the PCR [23–26]. Other methods improve the ease and efficiency of amplification by introducing specific modifications to the cycling conditions, as in site-finding PCR and touchdown PCR [27, 28]. Still other methods use restriction enzymes to fragment the DNA at defined domains thereby enabling modifications to the DNA terminus via adaptor ligation and/or template circularization steps, as in inverse, vectorette, T-linker and ligation-mediated PCR [29–31]. More recently there’s been a move towards the use of probes and sequence-capture techniques to address problems with non-specific amplification [32, 33]. With so many niche methods to address the same question, it’s not surprising that insertion site characterization itself is still seen as a difficult niche pursuit, and most applications are done long after establishment of stable lines manifesting a particularly interesting phenotype.

Our aim is not to discount any of the methods listed above but rather to offer an alternative method specifically intended to be used early in the transgenic process without requiring specialized training or equipment. First, this method does not require any preprocessing or pre-treatment of the gDNA prior to library prep. Template concentrations requirements for this method are also no more stringent than what is needed for a standard PCR reaction. Additionally, all steps leading up to sequencing involve materials that are accessible to most labs (i.e. reagents for DNA extraction, custom primers, thermal cycler, gel electrophoresis). Because sequencing primers and indices are compatible with any Illumina-based platform, multiple PCR reactions and samples can be pooled together into a single multiplex library and submitted for paired-end 150 bp NGS sequencing to any service provider so long as the sample sequencing depth is sufficient. Although it is reasonable to assume that deep sequencing of different PCR reactions and genome-walking primers can potentially yield more information and detection of rare insertions, here we have demonstrated that by pooling the four different genome-walking reactions together prior to indexing, we were able to reduce the total number of sequencing reactions to two per T_1_ individual (one left-border and one right-border reaction) without compromising our ability to detect the insertion sites that were verified in this study.

Despite not being able to identify the location of the OGC insertion in line #1416 in this sequencing run, we feel that this result highlights a key advantage to performing insertion site screening at an early stage. Having knowledge of any insertion site grants the researcher the option to eliminate the unwanted or uncharacterized insertions either through backcrossing to a wildtype or through normal segregation. It should also be noted that the T_2_ generation plants evaluated here were selected for both antibiotic resistance markers, however, it would be possible to generate a single-copy line that is homozygous for OCP1 in the absence of selection. Moving forward in this way would likely be the easiest alternative, rather than attempting to track down every insertion in a given line, but is not the only option. Among a number of factors that could have influenced our ability to detect the OGC insertion in this line, including a possible truncation or deletion of border sequences during transgene integration, one experimental factor that deserves consideration is the genome walking primer sequences that were used. Here, the four different genome walking primer motifs were selected for the purpose of testing this approach and, although the selection procedure was systematic, it is still possible that none of our motifs are proximal to the OGC insertion site, or at least within a range that is amplifiable. This possibility seems more plausible if you consider that three of the four primer motifs used here had a GC-content of 66.67% and, although there is some debate on this in the literature, several studies that have analyzed sequence characteristics of insertion sites have found a preference for integration in AT-rich regions [34–36]. In this case, and if it is in the interest of the researcher to continue pursuing an insertion that was not detected in the sequencing, our recommendation would be to simply apply the same technique using another set of genome walking primer motifs.

## Conclusions

Here, we present an accessible method for identifying the genomic location of an inserted transgene for multiple lines and transgene constructs at a time. Having this information at an early stage during transgenic line development is particularly useful for applications involving integration of multi-construct or “stacked” lines by accelerating the process of generating a stable, homozygous line. In practice this requires a substantial investment of time and resources into growing multiple independent lines, followed by selection and segregation of each line’s progeny until segregation ratios are in accordance with what is expected from a homozygote. If multiple transgenes were inserted into a line, homozygosity for each and all insertion sites can only be determined with certainty using whole genome sequencing. However, if transgene insertion sites are defined in the T_1_ generation, it is possible to obtain homozygous lines in as early as the T_2_ generation through simple PCR genotyping of seedlings, with similar confidence to these more intensive methods.

Aside from gains in time and resources, there are other obvious (and arguably of equal importance) benefits to obtaining such information which should be considered here as well. Decades of research involving the use of *Agrobacterium* for gene delivery have demonstrated that T-DNA insertion events are rarely precise and can sometimes lead to unintended chromosomal rearrangements, translocations, incorporation of bacterial vector backbone sequence, T-DNA duplications and inversions, and other T-DNA sequence modifications. Previous studies have reported a higher success rate of identifying insertions adjacent to the T-DNA left-border sequence and in general, the right-border/plant genomic junction is more difficult to recover. This phenomenon of finding genomic sequences associated with one T-DNA border but not the other is common, especially among lines in the *Arabidopsis* T-DNA mutant collections [13–15, 21]. A subset of these lines have been characterized more thoroughly in recent years and their findings suggest that this phenomenon may in part be due to complex rearrangements within the plant genome or the T-DNA itself, leading to dissociation of sequence continuity between left- and right-border flanking sequences [14]. In some cases, these alterations occurred without any disruption to the phenotype or segregation pattern [12, 13] and therefore, would likely go unnoticed until further characterization. In other cases, phenotypic consequences were incurred but only in later generations. For these reasons, transgenic lines containing a single and fully-intact copy of each transgene are considered more desirable as they are more likely to exhibit normal segregation behavior and stable expression characteristics [37] as opposed to those with many insertions and a phenotype that is potentially confounded by unknown genetic effects or irregularities [14].

Early determination of insertion sites and transgene copy numbers enable a more information-based approach to selecting transgenic plants and identifying parental lines harboring insertions at known loci with predictable segregation behavior prior to propagation and characterization.

## Methods

### Genetic material

Wildtype *Camelina sativa* (cultivar Calena) plants were grown in a 1:1 mixture of soil/sand (Fafard Sunshine Mix #8) under long day conditions (16-h light/8-h dark) at 22 °C until onset of flowering. Two separate cultures of *Agrobacterium tumefaciens* strain GV3101 were prepared containing each of the two plasmids were grown, then mixed together in a 1:1 ratio and used to inoculate plants via resuspension in 5% sucrose plus 0.02% Silwet L-77 and vacuum infiltration of flowers. Seeds harvested from transformed plants were germinated on 0.5 × MS, 0.8% agar and 1% sucrose plates supplemented with 30 μg/mL hygromycin and 25 μg/mL phosphinothricin for antibiotic selection.

Seeds of the *A. thaliana* CIF1 mutant clone GK-269G12-015,062 [GenBank Accession: AL942877] were obtained from the Nottingham Arabidopsis Stock Centre (NASC, http://arabidopsis.info/) and grown under long day conditions (16-h light/8-h dark) at 22 °C.

Genomic DNA was extracted from leaf tissue using the CTAB extraction method [38] and quantified on a NanoDrop1000 spectrophotometer. DNA from transgenic *C. sativa* plants were screened via PCR to confirm positive integration of both transgenes using cassette-specific primers. Likewise, the *A. thaliana* T-DNA insertion line was screened using the primer sequences provided by the GABI-Kat collection. DNA concentrations were diluted to 30 ng/ul prior to library preparation.

### Primer design

#### Genome-walking primer selection

Primers used for semi-random amplification of unknown flanking regions were designed following the methodology described in Kalendar et al. 2019 [17]. As the frequency of primer binding sites can vary across the genome and influence the probability of transgene detection, primers should be selected to bind with just enough frequency in the genome to enable pairing with the transgene-specific primer at whatever site the TDNA is inserted, while minimizing background amplification. Ten primers were selected on the basis of average distance between neighboring palindromic sites in the *Arabidopsis* genome [17]. Specifically, we focused on motifs which were below the overall average distance to ensure genome “coverage” was sufficient but not excessive—i.e. with moderate frequency. The unique nucleotide sequences were selected based on the frequency of motif occurrence in the *Camelina sativa* genome. FastPCR [39] was used to predict the number of PCR amplicons between 30 bp and 2 kb that could be obtained using 6 bp primer motifs along with 10 leading degenerate bases (10 degenerate + 6 bp). Based on *in-silico* PCR and PST method recommendations, we selected a set of four motifs that resulted in a moderate number of random PCR amplicons compared to all other tested primer motifs (see Additional file 1 for more details). In addition, primers contained a universal 5’ Illumina adapter-linking sequence.

### Transgene-specific primer design

Two transgene-specific primers each were designed for the left and right borders. Sequences were selected to have a high melting temperature and total length above 29 bp in order to increase specificity for the target template. All of the transgene-specific primers bind in the region of the plasmid between the T-DNA border repeats and the transgene (or marker gene) regulatory sequences. The innermost (relative to the transgenic insert) primer (LB1 and RB1) was designed to bind 200-250 bp from the T-DNA border repeat whereas the nested, outermost primer (LB2 and RB2) binds closer to the T-DNA border, between 100 and 150 bp. Nested primers included an overhang sequence at the 5’ end to enable attachment of indexing primers for sequencing. The T-DNA border regions where the transgene-specific primers bind are highly conserved across plant transformation vectors. The 'transgene-specific' primers are therefore most appropriately viewed as specific to the T-DNA vector relative to the plant genome, but agnostic to variation between the OGC and OCP1 transgenes they contain. This property allowed the same transgene-specific primer sets to be used for amplification of both transgenes in the example application, and potentially for future work without modification.

To verify primer efficiency and specificity to the intended binding location, primers were tested in a PCR with a range of plasmid DNA concentrations (0.3 ng/ul, 10 ng/ul, and 30 ng/ul) and each of the four genome walking primers separately. Amplification was observed for all three template concentrations and band intensity increased with increasing concentration. High intensity bands were extracted and Sanger sequenced to confirm that nested primers accurately amplify the left- and right-border target regions.

### Index and adapter sequences

Sequencing primers were designed in the style of Illumina TruSeq HT dual-index primers. The primers contain P5 and P7 sequences at the 5’ end to enable binding of amplicons to an Illumina flow cell. The P5/P7 region is followed by an 8-bp index sequence as well an adapter sequence that is complementary to the primer overhang sequences used to target the T-DNA border (Read 2) and the universal adapter sequence in the genome walking primers (Read 1). With 20 total primers, we were able to tag 96 samples with unique dual-index combinations to be sequenced in the same run. Primer sequences are provided in Additional file 3.

### Library preparation

Sequencing libraries were prepared using three sequential PCR steps (Fig. 1a-c). For each transgenic line, two PCR reactions were performed using two nested transgene-specific reverse primers (LB1 & LB2 or RB1 & RB2) and a genome walking primer (PST1, PST2, PST3, PST4) or universal adapter (PST0). A final PCR step was included to attach barcodes and adapters to amplicons to allow for high-throughput screening of multiple lines via next generation sequencing.

### PCR step 1: Semi-random amplification

Genome walking primers were used in conjunction with a border-specific primer to amplify genomic sequence proximal to the transgene integration site. Separate PCR reactions were performed for left and right borders. For each line ~ 30 ng of genomic DNA was used as template in the following 25 μl reaction: 12.5 μl of GoTaq G2 Hot Start Master Mix, 0.2 μM of the transgene specific primer (LB1 or RB1), and 0.5 μM of genome walking primer.

Thermal cycling conditions were selected based on recommendations described in Kalendar et al. [16, 17]. The thermal profile consists of two stages: a linear phase which uses a high annealing temperature to favor binding of the transgene-specific primer for initial template formation, and an exponential phase at a lower annealing temperature to favor binding of random primers to the transgene-containing template. In theory, genome-walking primers do not bind at all during the first phase, resulting in an addition of 1 × the starting number of transgene insertion-containing fragments every cycle due solely to binding of the transgene-specific primer. At the end of the linear phase, transgene-containing fragments are expected to be enriched at ~ 19 × higher than background. Specific conditions were as follows: Initial denaturation for 3 min at 95℃ followed by linear amplification for 18 cycles at 95℃ for 15 s, 70℃ for 20 s, and 1-min extension at 72℃, and continued to the exponential phase for 18 cycles with 95C denaturation for 15 s, annealing at 50℃ for 15 s, extension for 1 min at 72℃, and then a final extension at 72℃ for 5 min. The resulting PCR products were diluted 1:5 to be used in the subsequent PCR.

### PCR Step 2: Nested PCR for transgene product enrichment

A second PCR was performed to enrich for transgene-containing targets using the outermost transgene-specific reverse primer with a 5’ overhang sequence for adapter attachment. This step also reduces the total length of the amplicon, thereby increasing the probability of obtaining a full read spanning the junction. A 25 μl PCR reaction was prepared as outlined in Step 1 with the following changes: 2 μl of the diluted PCR product from Step 1 were used as template, 0.2 μM of forward primer (PST0) containing only the conserved portion of the genome walking primers, and 0.2 μM of the transgene-specific primer (LB2 or RB2).

The second PCR is a two-step process where annealing and extension are combined into a single high temperature step to favor binding of the nested transgene-specific primer. Thermal cycling conditions were as follows: Initial denaturation for 3 min at 95℃ followed by 40 cycles of 95℃ denaturation for 15 s and annealing/extension at 72℃ for 1:30 min, with a final extension of 5 min at 72℃. PCR products were run on a QIAxcel automatic capillary electrophoresis gel (QIAxcel DNA Screening Kit, Qiagen) and visualized using the QIAxcel ScreenGel Software (QIAxcel Advanced System, Qiagen) to confirm that products were successfully amplified before proceeding to index attachment. Gel images are also provided in supplemental information (Additional file 4, Figures S1-S4).

### PCR Step 3: Sample indexing and Illumina adapter attachment

Sequencing adapters and barcode indices were incorporated in a final PCR reaction containing 5ul of the PCR product from step two. At the end of step 1, the genomic-walking end of the PCR amplicons contain the 5’ overhang sequence compatible with i5 index and adapter primers and at the end of step 2, the transgene-specific primer containing a 5’ overhang compatible with i7 index and adapter primers is incorporated onto the transgene-specific end. In step 3, overhang sequences are utilized in a single PCR reaction to attach a unique index and a P5 or P7 sequencing adapter to each end of the amplicon. Full primer sequences are available in Additional file 3. A dual-indexing strategy was used to allow for identification of each border, line, and PCR reaction. Reactions that were prepared with a single random primer or all four primers in PCR step one received unique barcodes whereas reactions containing pairs of random primers were combined into a single library with one unique index.

Thermal cycling conditions were as follows: Initial denaturation for 2 min at 95℃ followed by 15 cycles at 95℃ for 15 s, 65℃ for 30 s, 72℃ for 1:30 s, and final extension for 5 min at 72℃. PCR products were run on a QIAxcel automatic capillary electrophoresis gel (QIAxcel DNA Screening Kit, Qiagen) and visualized using the QIAxcel ScreenGel Software (QIAxcel Advanced System, Qiagen) to confirm that the length of PCR products followed expectation and had increased due to adapter incorporation. Gel images are also provided in supplemental information (Additional file 4, Figures S1-S4).

### Library purification, quantitation, and normalization

Indexed PCR reactions were combined into a single multiplex library and purified using an Invitrogen PowerSnap electrophoresis device and a E-Gel CloneWell II 0.8% pre-cast agarose gel. The pooled library was loaded along with an E-Gel 50 bp DNA ladder and products were extracted over the course of migration in increments of 100 bp (from 150 bp to 1.5 kb) and binned according to size into separate tubes. The concentration of purified products were quantified on a Qubit fluorometer using the dsDNA high-sensitivity assay kit and used to determine the molar concentration of products for each size bin. Molarity calculations were used to dilute each bin to 1 nM and then pooled together in equimolar quantities into a single (normalized) sequencing library.

### Sequencing

Sequencing was performed on an Illumina iSeq100 benchtop sequencer to obtain 2X150bp paired-end reads. Library loading concentration was experimentally determined and calibrated based on the average molarity of the final pooled library along with a 5% PhiX spike-in for increased diversity. Raw sequencing data was demultiplexed using the Local Run Manager FASTQ Analysis Module in the iSeq100 Local Run Manager software.

### Data processing

A total of 3.3 million read pairs were obtained for the three transgenic lines and wildtype samples combined. Of these, approximately 1.35 million reads corresponded to PCRs for the left border and 2 million reads for the right border. Reads were filtered for adapter contamination using Trimmomatic with default parameter settings and quality was assessed using FASTQC [40]. Although a large fraction of reads was obtained from wildtype samples (~ 34%), the majority appeared to be adapter dimers and represented a negligible proportion after adapter filtering (~ 0.007%). The number of reads remaining for the transgenic samples were much higher (> 350,000 per line) after adapter removal.

Reads were mapped separately based on the library design. Reads corresponding to the border sequence (Read 2) were aligned to a reference containing both T-DNA vector sequences using HISAT2 [41] with the hard clipping function disabled and all other default parameter settings. The names of positively mapped reads (MAPQ > 10) were then used to identify the corresponding mate (Read 1) and aligned to the full T-DNA reference sequence. Sequence alignment map (SAM) flags were used to identify clipped sequences, or regions that did not align to the reference and were a minimum of 20 bp in length, using the samextractclip tool in JVarkit [42]. Specifically, we were interested in 5’-clipped regions from the left border aligned reads, and for the right border alignments, the 3’-clipped ends. All reads were combined into a single file for each transgenic and wildtype plant and duplicated sequences were removed using fastq-uniq in fastq-tools to reduce file size. Files were converted to FASTA format prior to use in BLAST + command line.

### BLAST query

For each transgenic line, the full list of clipped and unmapped sequences obtained from the left- and right-border libraries were queried against the GenBank genomic reference sequence database for Camelina sativa (GCF_000633955.1) using the blastn application in BLAST + command line toolkit [43]. Search parameters were set to default values with the exception of percent identity and e-value, which were set to 95% and 1e-10 respectively, in order to restrict query results to only high identity matches.

## Supplementary Information

**Additional file 1:** Description of motif selection process for genome walking primers **Table S1.**

**Additional file 2:** **Table S2.** List of all plant genomic primer sequences used for T-DNA flanking PCRs.

**Additional file 3.** Primers for indexing and sequencing adapter attachment.

**Additional file 4:** Capillary gel electrophoresis results for library prep and additional T2 zygosity PCR results. **Figures S1-S4.** Side-by-side gel images of amplified products obtained from the nested PCR (step 2) before and after (step 3) indexing for every line. PCR step 1 results in a broad range of low-intensity bands and in most cases, strong, specific bands are not detected until PCR step 2. For this reason, it is important to note that PCR success or failure should not be evaluated based on results of the PCR step 1. **Figure S5.** Gel electrophoresis results of the T2 zygosity screening PCRs for the OGC transgene in line #1415. **Figures S6-S7.** Gel electrophoresis results of the two T2 zygosity screening PCRs for the OCP1 insertion site in line #1416 showing the non-Mendelian segregation of the transgene on chromosome 8. **Figures S8-S9.** Full length gel electrophoresis results of the PCRs shown in Figure 4. Results of both PCRs were used to assess T2 zygosity at the OCP1 insertion site in line #1416 on chromosome 15.

## Data Availability

The datasets supporting the conclusions of this article and sequences for all primers used in this study are included within the article and its additional file(s). All sequence data generated and used for the current study are available in the DRYAD repository under https://doi.org/10.5061/dryad.p8cz8w9sg.

## Abbreviations

T-DNA: The sequence of DNA sequence delivered and integrated during transformation
LB: T-DNA left-border region
RB: T-DNA right-border region
PCR: Polymerase chain reaction
PST: Palindromic sequence-target
gDNA: Genomic DNA
SNP: Single nucleotide polymorphism
BLAST: Basic local alignment search tool
