# Supplementary material for: High-throughput detection of T-DNA insertion sites for multiple transgenes in complex genomes
Source: BMC Genomics. 2022 Oct 5;23:685. doi: 10.1186/s12864-022-08918-6 (PMC9533571; doi:10.1186/s12864-022-08918-6)
Supplement: Supplementary file 1 — Additional file 1: Description of motif selection process for genome walking primers Table S1. [file 12864_2022_8918_MOESM1_ESM.docx]

**Additional file 1: Description of motif selection process for genome walking primers.**

PST primer 6-bp motifs were first selected on the basis of frequency of occurrence and average distance between neighboring motifs in the Arabidopsis genome based on the Supplementary Dataset provided in Kalendar et al. 2019. Specifically, we focused on motifs which were below the average distance across all motifs (<13,350) for the purpose of having an increased probability of the motif occurring near a T-DNA border and within a range that is amplifiable and able to be sequenced on a short-read platform. From the remaining motifs, we grouped the remaining motifs into bins of 1 kb with distances spanning from 1,000 to 12,000 bp and selected one motif within each bin to use for initial primer testing in Camelina.

In addition to length and distance between neighboring motifs, factors such as motif binding frequency and product formation potential are also important factors to consider during the selection process. In order to estimate the performance of the selected primer motifs in Camelina, a virtual PCR was performed using the *in silico* PCR tool from the FastPCR software [1,2]. Ten degenerate bases were added to the 5’ end of the selected motifs to create the full 16-bp genome walking primer sequence (without the 5’ overhang). Genome-walking primers were used as input for an *in silico* PCR to predict the number of theoretically obtainable PCR products from each Camelina chromosome. Run parameters were restricted to product sizes below 2 kb and allowed for 1-nt mismatch in the 3’ end. PCR results were compiled for all of the selected motifs to compare how the binding frequency and expected number of products differ across the whole genome (see below table). For each primer, we calculated the total number of predicted PCR products and standard deviation was summarized across the whole genome. To make comparisons at the chromosome-level, we also calculated mean, median, Q30, and Q70 summary statistics for each chromosome separately. With the specific aim of selecting motifs with a moderate number of predicted PCR products, we identified primers whose predictions were within the Q30 and Q70 range at the scale of the whole genome, the standard deviation across all chromosomes, and within individual chromosomes (denoted in green boxes in the table below). Primers PST4, PST5, PST6, and PST10 were more consistently within this quartile range across all metrics and were therefore selected as the primary primer motifs for library preparation.

**References**

1. Kalendar R, Samuilova O, Ivanov KI 2017. FastPCR: an in silico tool for fast primer and probe design and advanced sequence analysis. Genomics, 109(4-5): 312-319. DOI:10.1016/j.ygeno.2017.05.005
2. Kalendar R, Lee D, Schulman AH 2011. Java web tools for PCR, in silico PCR, and oligonucleotide assembly and analysis. Genomics, 98(2): 137-144. DOI:10.1016/j.ygeno.2011.04.009

**Table S1.**

|  |  | **Number of PCR products predicted (between 30bp-2kb)** | | | | | | | | | | | | | | | | | | | |  |  |
| --- | --- | --- | --- | --- | --- | --- | --- | --- | --- | --- | --- | --- | --- | --- | --- | --- | --- | --- | --- | --- | --- | --- | --- |
| **PST**  **primer** | **Sequence** | **chr**  **1** | **chr**  **2** | **chr**  **3** | **chr**  **4** | **chr**  **5** | **chr**  **6** | **chr**  **7** | **chr**  **8** | **chr**  **9** | **chr**  **10** | **chr**  **11** | **chr**  **12** | **chr**  **13** | **chr**  **14** | **chr**  **15** | **chr**  **16** | **chr**  **17** | **chr**  **18** | **chr**  **19** | **chr**  **20** | **Genome total** | **Standard**  **deviation** |
| **PST1** | NNNNNNNNNNAAGCTT | 12908 | 16999 | 16369 | 18581 | 19660 | 14879 | 18290 | 15920 | 23122 | 14605 | 28627 | 19518 | 13885 | 17778 | 18254 | 16172 | 20656 | 12692 | 15532 | 17219 | 351666 | 3689.810337 |
| **PST2** | NNNNNNNNNNAGATCT | 7773 | 9941 | 8813 | 9513 | 11216 | 8167 | 10883 | 9614 | 12461 | 8225 | 15531 | 15397 | 8007 | 9782 | 10055 | 8835 | 10851 | 6780 | 8576 | 10444 | 200864 | 2293.734087 |
| **PST3** | NNNNNNNNNNGATATC | 3049 | 3566 | 3717 | 4062 | 4543 | 3433 | 4438 | 3597 | 4827 | 3314 | 6714 | 4242 | 3116 | 4352 | 4015 | 3815 | 4569 | 2830 | 3661 | 4001 | 79861 | 842.0711606 |
| **PST4** | NNNNNNNNNNGTTAAC | 2069 | 3026 | 2552 | 2856 | 3065 | 2586 | 3233 | 2303 | 3490 | 2250 | 4591 | 2963 | 2218 | 2775 | 2867 | 2780 | 3150 | 1829 | 2374 | 2575 | 55552 | 600.2232392 |
| **PST5** | NNNNNNNNNNCCATGG | 3266 | 2715 | 2520 | 2582 | 3313 | 2145 | 2713 | 2853 | 3062 | 2705 | 4602 | 3009 | 2468 | 2851 | 3384 | 2475 | 3375 | 1839 | 2290 | 2796 | 56963 | 582.5908806 |
| **PST6** | NNNNNNNNNNCTCGAG | 1604 | 2465 | 1836 | 2608 | 4337 | 1976 | 2494 | 2390 | 3000 | 1949 | 3987 | 3059 | 1595 | 2526 | 2603 | 1811 | 2697 | 1502 | 1903 | 2406 | 48748 | 747.6928303 |
| **PST7** | NNNNNNNNNNCACGTG | 846 | 898 | 1067 | 1171 | 1237 | 1064 | 1250 | 991 | 1531 | 1140 | 1951 | 1179 | 986 | 1062 | 1008 | 1257 | 1163 | 790 | 893 | 1037 | 22521 | 257.9847966 |
| **PST8** | NNNNNNNNNNGCATGC | 1068 | 1832 | 1326 | 1728 | 2143 | 1281 | 1540 | 1478 | 2430 | 1399 | 2724 | 2350 | 1229 | 1767 | 1834 | 1378 | 2029 | 1000 | 1390 | 1811 | 33737 | 467.3527097 |
| **PST9** | NNNNNNNNNNGCTAGC | 688 | 976 | 770 | 780 | 1427 | 781 | 876 | 892 | 1598 | 762 | 1699 | 1213 | 739 | 713 | 1550 | 829 | 1763 | 507 | 807 | 1449 | 20819 | 391.8598669 |
| **PST10** | NNNNNNNNNNGTCGAC | 2097 | 1719 | 2542 | 1528 | 2267 | 2212 | 1963 | 1696 | 2987 | 2429 | 2708 | 2594 | 1516 | 1917 | 2484 | 1862 | 2508 | 1884 | 1414 | 2048 | 42375 | 438.7110157 |
|  |  |  |  |  |  |  |  |  |  |  |  |  |  |  |  |  |  |  |  |  |  |  |  |
|  | **mean** | 3536.8 | 4413.7 | 4151.2 | 4540.9 | 5320.8 | 3852.4 | 4768 | 4173.4 | 5850.8 | 3877.8 | 7313.4 | 5552.4 | 3575.9 | 4552.3 | 4805.4 | 4121.4 | 5276.1 | 3165.3 | 3884 | 4578.6 | 91310.6 | 1031.203092 |
|  | **median** | 2083 | 2590 | 2531 | 2595 | 3189 | 2178.5 | 2603.5 | 2346.5 | 3031 | 2339.5 | 4289 | 2986 | 1906.5 | 2650.5 | 2735 | 2168.5 | 2923.5 | 1834 | 2096.5 | 2490.5 | 52150 | 591.4070599 |
|  | **Q70** | 3200.9 | 3404 | 3367.5 | 3700.2 | 4481.2 | 3178.9 | 4076.5 | 3373.8 | 4425.9 | 3131.3 | 6080.4 | 3887.1 | 2921.6 | 3901.7 | 3825.7 | 3504.5 | 4210.8 | 2546.2 | 3274.9 | 3639.5 | 72991.6 | 813.7576615 |
|  | **Q30** | 1228.8 | 1752.9 | 1479 | 1588 | 2180.2 | 1489.5 | 1666.9 | 1543.4 | 2597.1 | 1564 | 2712.8 | 2423.2 | 1315.1 | 1812 | 2029 | 1507.9 | 2172.7 | 1150.6 | 1397.2 | 1882.1 | 36328.4 | 447.3035239 |
