# Supplementary material for: High-throughput detection of T-DNA insertion sites for multiple transgenes in complex genomes
Source: BMC Genomics. 2022 Oct 5;23:685. doi: 10.1186/s12864-022-08918-6 (PMC9533571; doi:10.1186/s12864-022-08918-6)
Supplement: Supplementary file 3 — Additional file 3. Primers for indexing and sequencing adapter attachment. [file 12864_2022_8918_MOESM3_ESM.docx]

**Additional file 3: Primers for indexing and sequencing adapter attachment.**

**Table S4. List of primer sequences used for multiplexing in PCR step 3.** List of primers sequences used in PCR step 3 to add unique barcodes to individual PCR reactions for multiplex sequencing. Nucleotides in brackets correspond to 8-bp indices and bold nucleotides mark the primer regions complementary to the 5’ overhang sequences in genome-walking and transgene-specific primers. Primers with i5 prefix associate with genome-walking primers and P5 sequencing adapter and correspond to read 1 in a 2x150bp sequencing run whereas primers with an i7 prefix associate with the transgene-specific end of the amplicon, the P7 sequencing adapter, and read 2.

| i5-D501 | AATGATACGGCGACCACCGAGATCTACAC[AGGCTATA]ACACTCTTTC**CCTACACGACGCTCTTCCGATCT** |
| --- | --- |
| i5-D502 | AATGATACGGCGACCACCGAGATCTACAC[GCCTCTAT]ACACTCTTTC**CCTACACGACGCTCTTCCGATCT** |
| i5-D503 | AATGATACGGCGACCACCGAGATCTACAC[AGGATAGG]ACACTCTTTC**CCTACACGACGCTCTTCCGATCT** |
| i5-D504 | AATGATACGGCGACCACCGAGATCTACAC[TCAGAGCC]ACACTCTTTC**CCTACACGACGCTCTTCCGATCT** |
| i5-D505 | AATGATACGGCGACCACCGAGATCTACAC[CTTCGCCT]ACACTCTTTC**CCTACACGACGCTCTTCCGATCT** |
| i5-D506 | AATGATACGGCGACCACCGAGATCTACAC[TAAGATTA]ACACTCTTTC**CCTACACGACGCTCTTCCGATCT** |
| i5-D507 | AATGATACGGCGACCACCGAGATCTACAC[ACGTCCTG]ACACTCTTTC**CCTACACGACGCTCTTCCGATCT** |
| i5-D508 | AATGATACGGCGACCACCGAGATCTACAC[GTCAGTAC]ACACTCTTTC**CCTACACGACGCTCTTCCGATCT** |
| i7-D701 | CAAGCAGAAGACGGCATACGAGAT[CGAGTAAT]GTGACTGGAGTTC**AGACGTGTGCTCTTCCGATCT** |
| i7-D702 | CAAGCAGAAGACGGCATACGAGAT[TCTCCGGA]GTGACTGGAGTTC**AGACGTGTGCTCTTCCGATCT** |
| i7-D703 | CAAGCAGAAGACGGCATACGAGAT[AATGAGCG]GTGACTGGAGTTC**AGACGTGTGCTCTTCCGATCT** |
| i7-D704 | CAAGCAGAAGACGGCATACGAGAT[GGAATCTC]GTGACTGGAGTTC**AGACGTGTGCTCTTCCGATCT** |
| i7-D705 | CAAGCAGAAGACGGCATACGAGAT[TTCTGAAT]GTGACTGGAGTTC**AGACGTGTGCTCTTCCGATCT** |
| i7-D706 | CAAGCAGAAGACGGCATACGAGAT[ACGAATTC]GTGACTGGAGTTC**AGACGTGTGCTCTTCCGATCT** |
| i7-D707 | CAAGCAGAAGACGGCATACGAGAT[AGCTTCAG]GTGACTGGAGTTC**AGACGTGTGCTCTTCCGATCT** |
| i7-D708 | CAAGCAGAAGACGGCATACGAGAT[GCGCATTA]GTGACTGGAGTTC**AGACGTGTGCTCTTCCGATCT** |
| i7-D709 | CAAGCAGAAGACGGCATACGAGAT[CATAGCCG]GTGACTGGAGTTC**AGACGTGTGCTCTTCCGATCT** |
| i7-D710 | CAAGCAGAAGACGGCATACGAGAT[TTCGCGGA]GTGACTGGAGTTC**AGACGTGTGCTCTTCCGATCT** |
| i7-D711 | CAAGCAGAAGACGGCATACGAGAT[GCGCGAGA]GTGACTGGAGTTC**AGACGTGTGCTCTTCCGATCT** |
| i7-D712 | CAAGCAGAAGACGGCATACGAGAT[CTATCGCT]GTGACTGGAGTTC**AGACGTGTGCTCTTCCGATCT** |

Oligonucleotide sequences © 2021 Illumina, Inc. All rights reserved.
