# Supplementary material for: High-throughput detection of T-DNA insertion sites for multiple transgenes in complex genomes
Source: BMC Genomics. 2022 Oct 5;23:685. doi: 10.1186/s12864-022-08918-6 (PMC9533571; doi:10.1186/s12864-022-08918-6)
Supplement: Supplementary file 4 — Additional file 4: Capillary gel electrophoresis results for library prep and additional T2 zygosity PCR results. Figures S1-S4. Side-by-side gel images of amplified products obtained from the nested PCR (step 2) before and after (step 3) indexing for every line. PCR step 1 results in a broad range of low-intensity bands and in most cases, strong, specific bands are not detected until PCR step 2. For this reason, it is important to note that PCR success or failure should not be evaluated based on results of the PCR step 1. Figure S5. Gel electrophoresis results of the T2 zygosity screening PCRs for the OGC transgene in line #1415. Figures S6-S7. Gel electrophoresis results of the two T2 zygosity screening PCRs for the OCP1 insertion site in line #1416 showing the non-Mendelian segregation of the transgene on chromosome 8. Figures S8-S9. Full length gel electrophoresis results of the PCRs shown in Figure 4. Results of both PCRs were used to assess T2 zygosity at the OCP1 insertion site in line #1416 on chromosome 15. [file 12864_2022_8918_MOESM4_ESM.pdf]

Additional file 4. Capillary gel electrophoresis results for library prep and additional T2 zygosity PCR results.

Figures S1-S4. Side-by-side gel images of amplified products obtained from the nested PCR (step 2) before and after (step 3) indexing for every line. PCR step 1 results in a broad range of low-intensity bands and in most cases, strong, specific bands are not detected until PCR step 2. For this reason, it is important to note that PCR success or failure should not be evaluated based on results of the PCR step 1.

Figure S5. Gel electrophoresis results of the T2 zygosity screening PCRs for the OGC transgene in line #1415.

Figures S6-S7. Gel electrophoresis results of the two T2 zygosity screening PCRs for the OCP1 insertion site in line #1416 showing the non-Mendelian segregation of the transgene on chromosome 8.

Figures S8-S9. Full length gel electrophoresis results of the PCRs shown in Figure 4. Results of both PCRs were used to assess T2 zygosity at the OCP1 insertion site in line #1416 on chromosome 15.

Figure S1

Camelina sativa
Line #1415

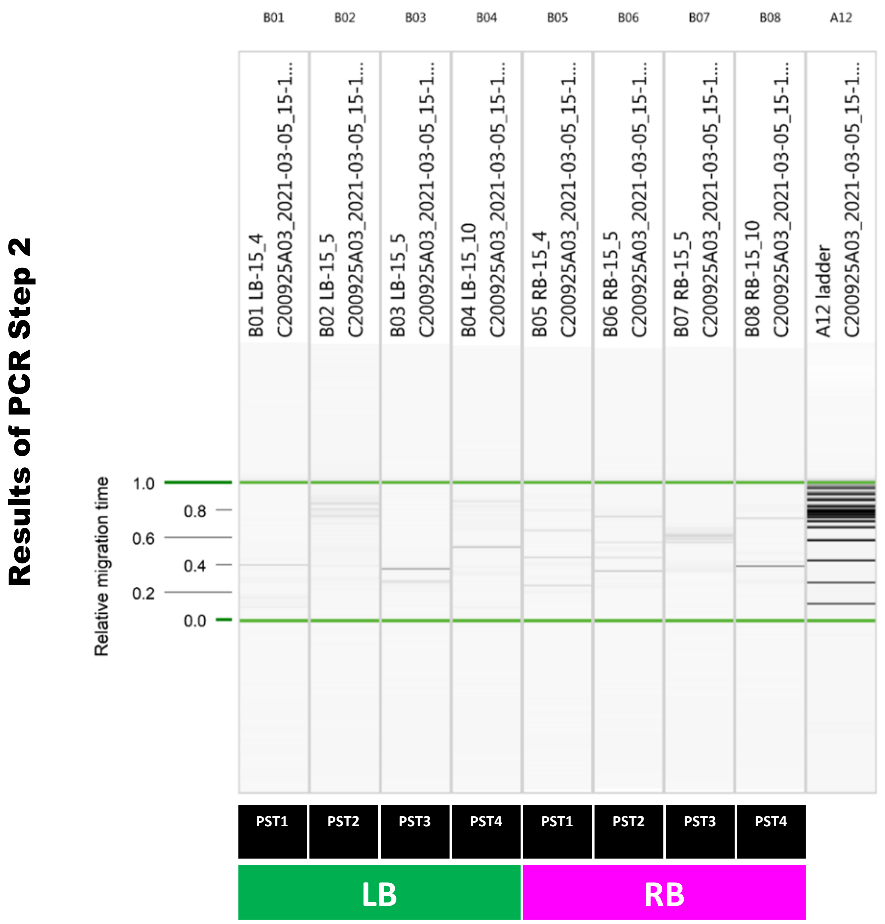

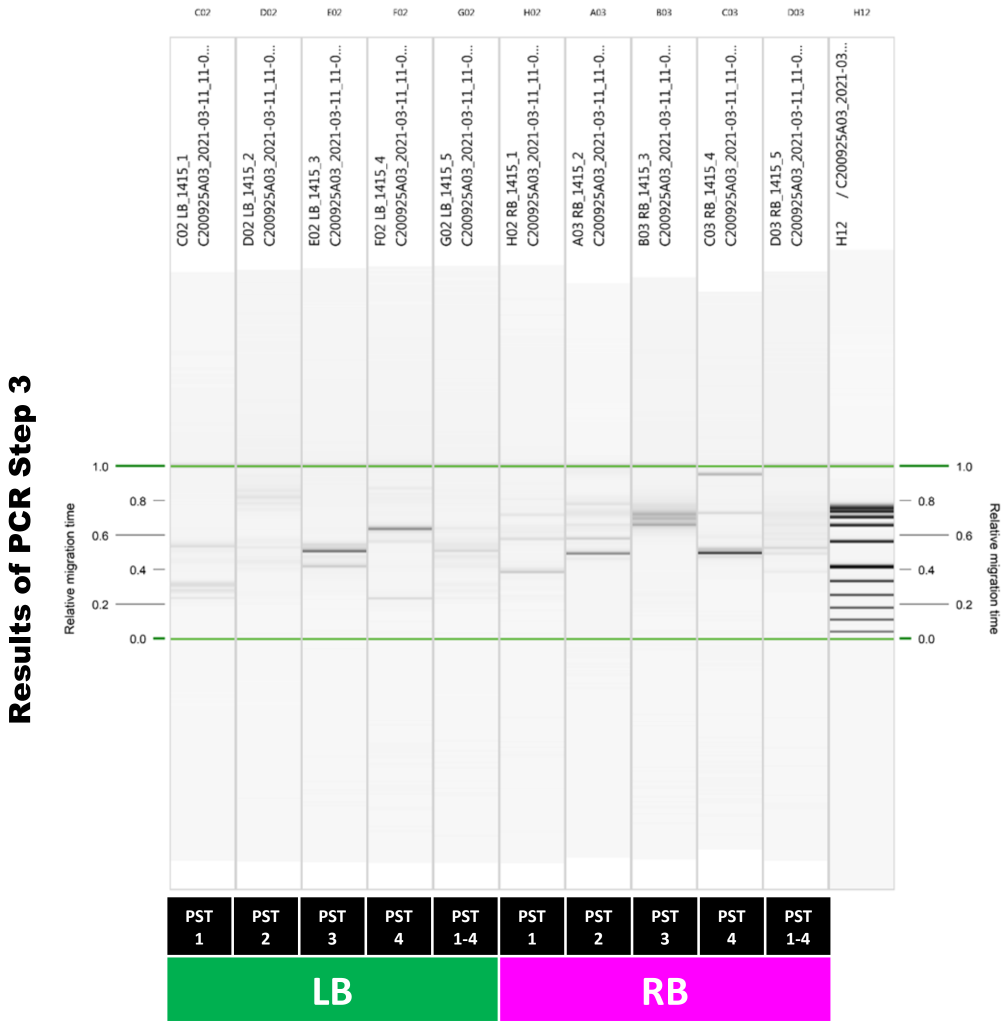

Figure S2

Camelina sativa Line #1416

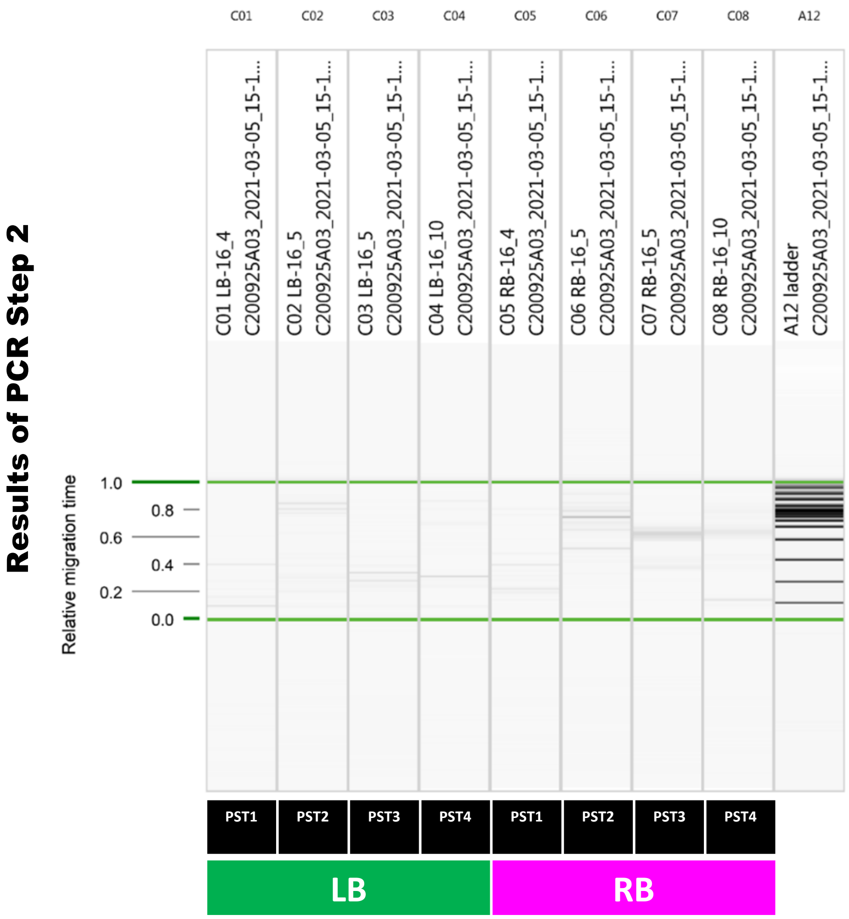

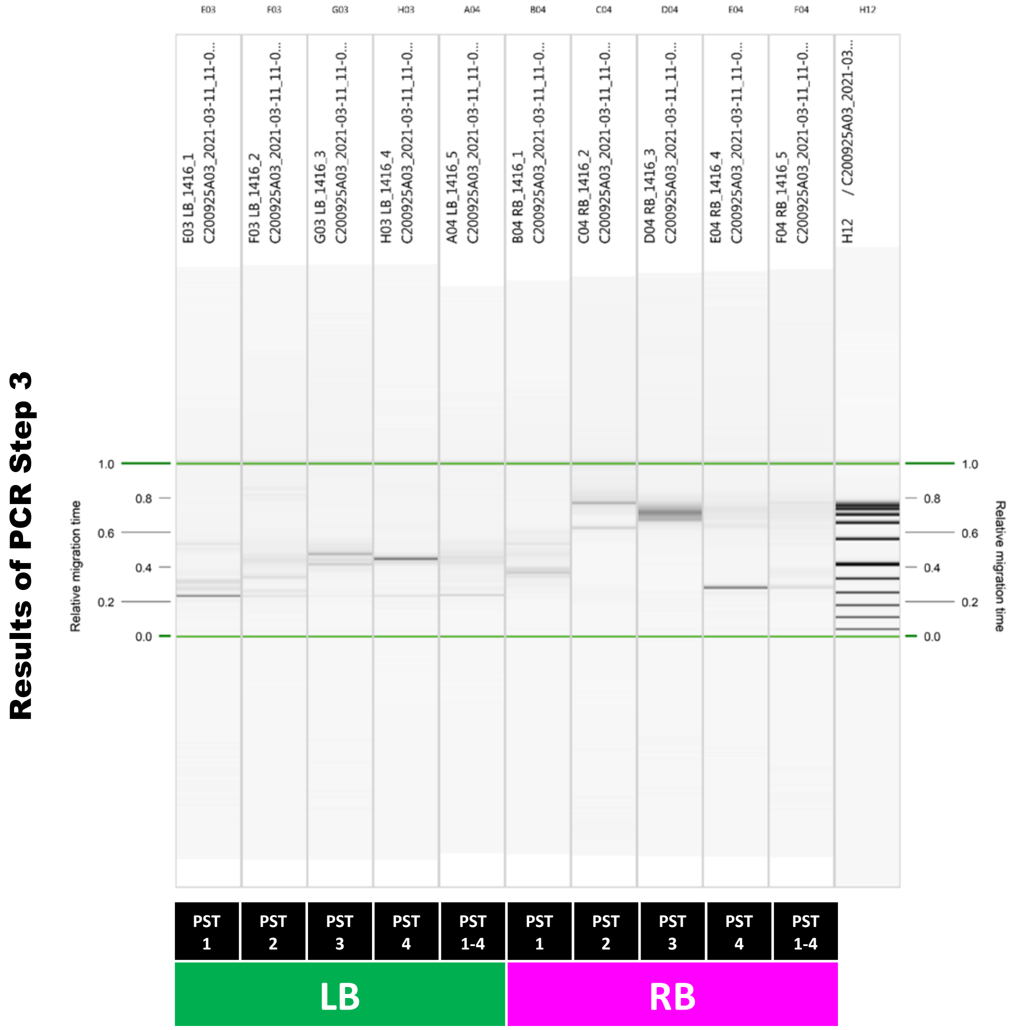

Figure S3

Arabidopsis GK-269g12

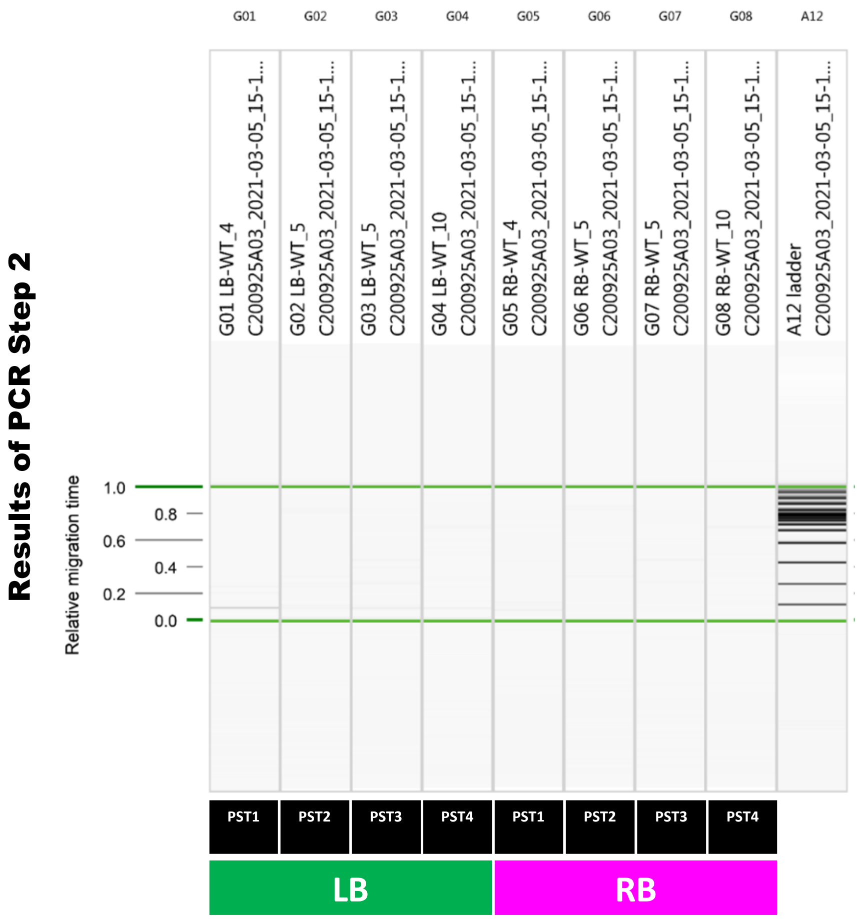

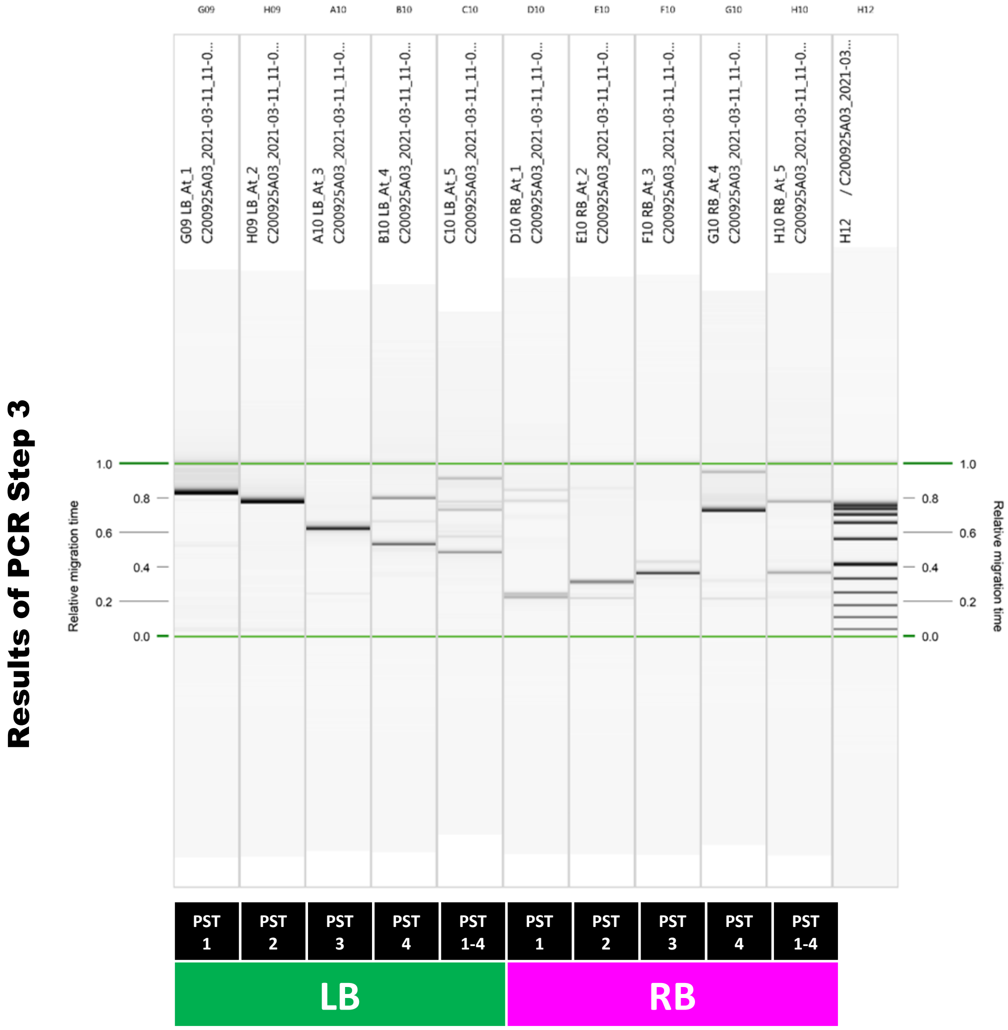

Figure S4

Camelina sativa
Wildtype

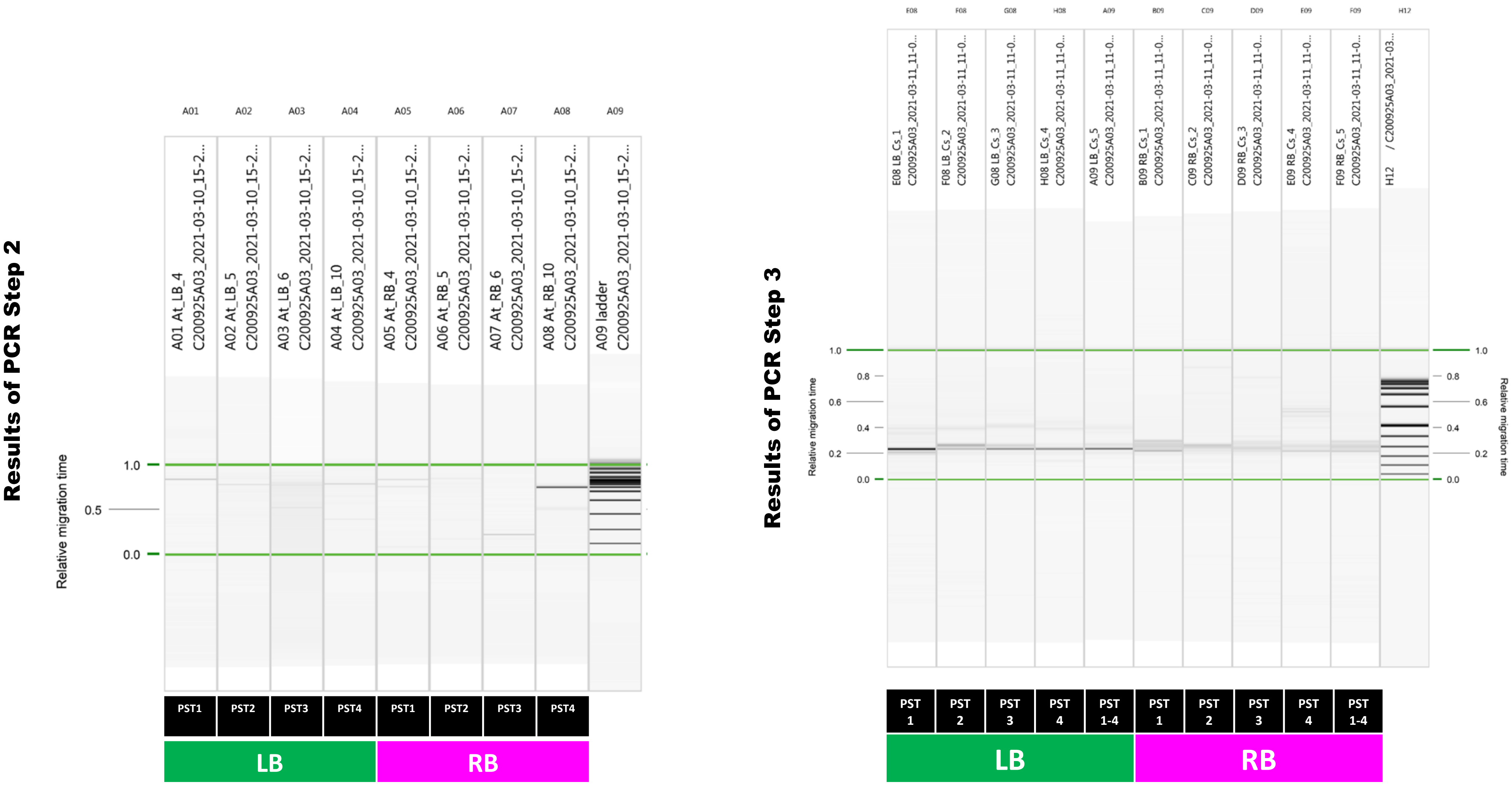

Figure S5

Camelina sativa
Line #1415
OGC Insertion at Chr5:9760996

Left-border

T-DNA anchored PCR A

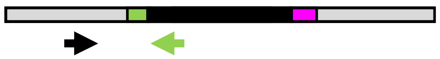

T-DNA flanking PCR B

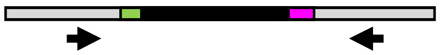

T2 zygosity PCR

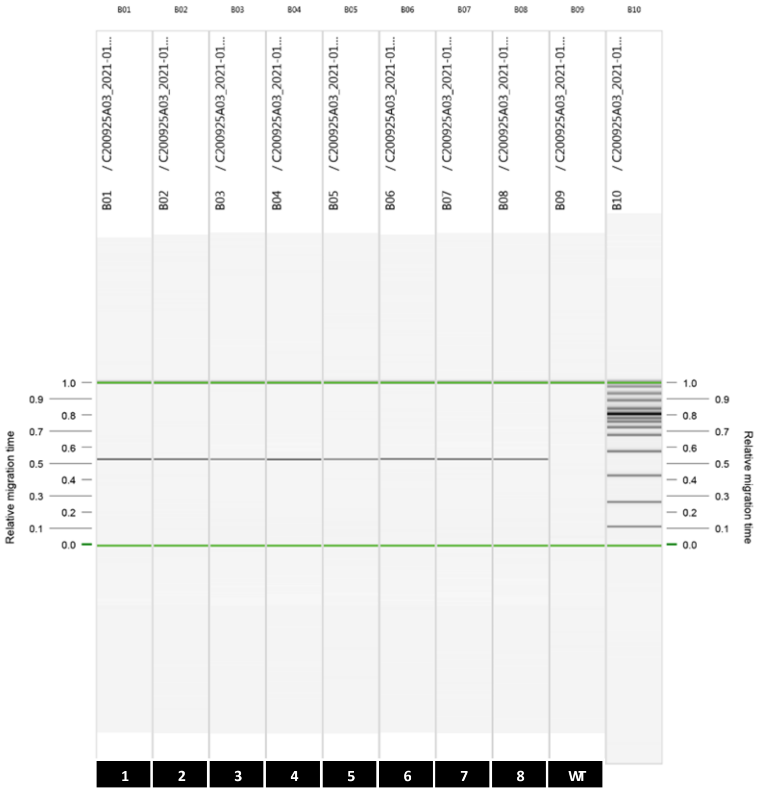

T2 plant #

T2 zygosity PCR

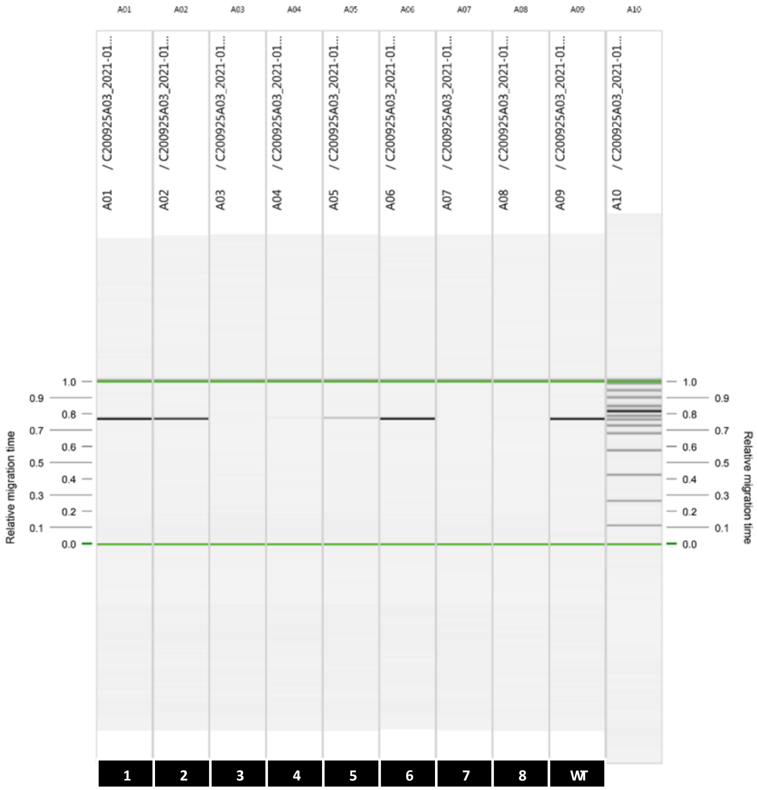

T2 plant #

Figure S6

Camelina sativa
Line #1416
OCP1 insertion at Chr8:24442089

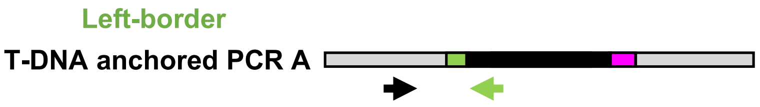

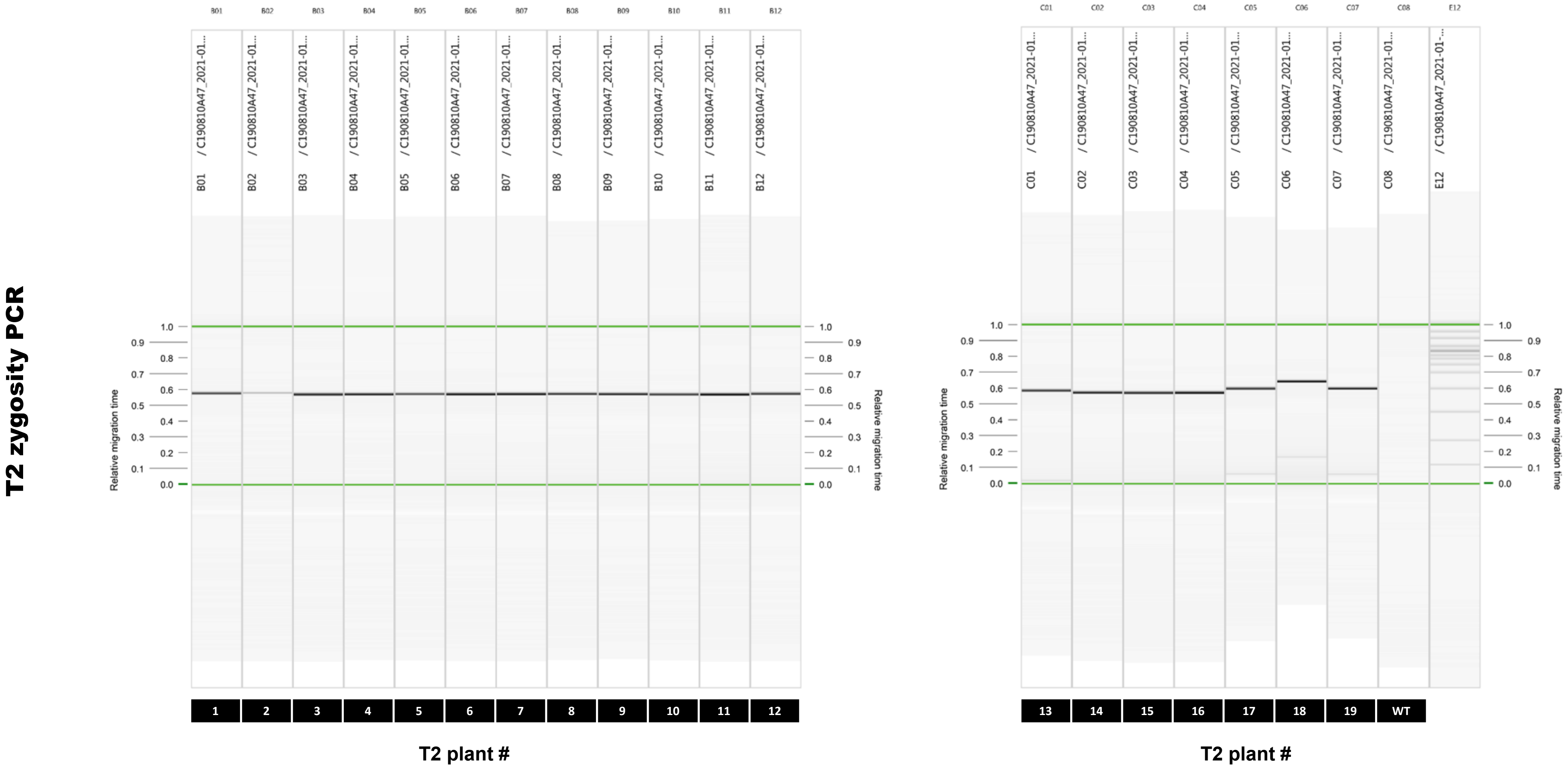

Figure S7

Camelina sativa
Line #1416
OCP1 insertion at Chr8:24442089

T-DNA flanking PCR B

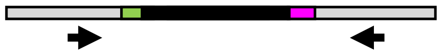

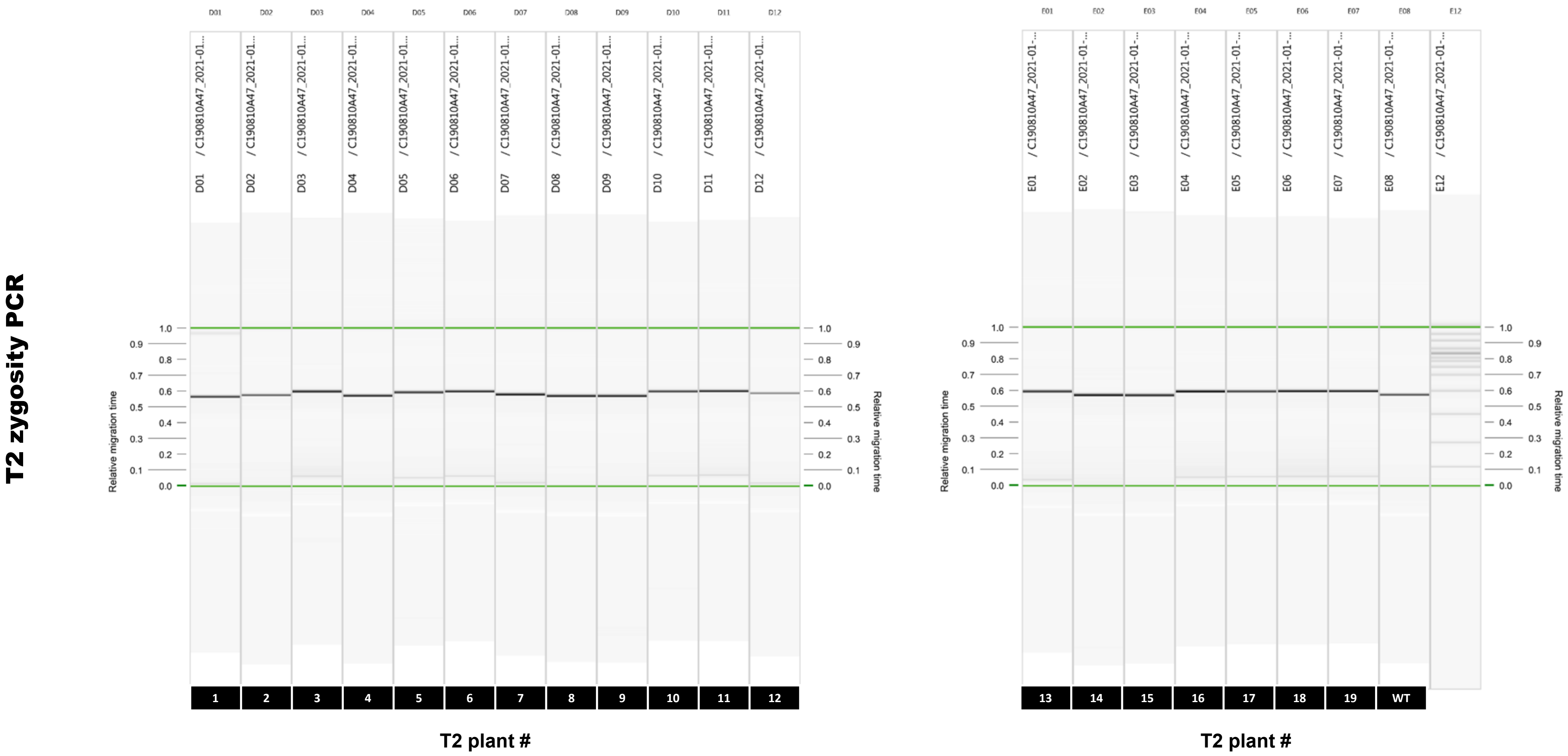

Figure S8

Camelina sativa
Line #1416
OCP1 insertion at Chr15:982591

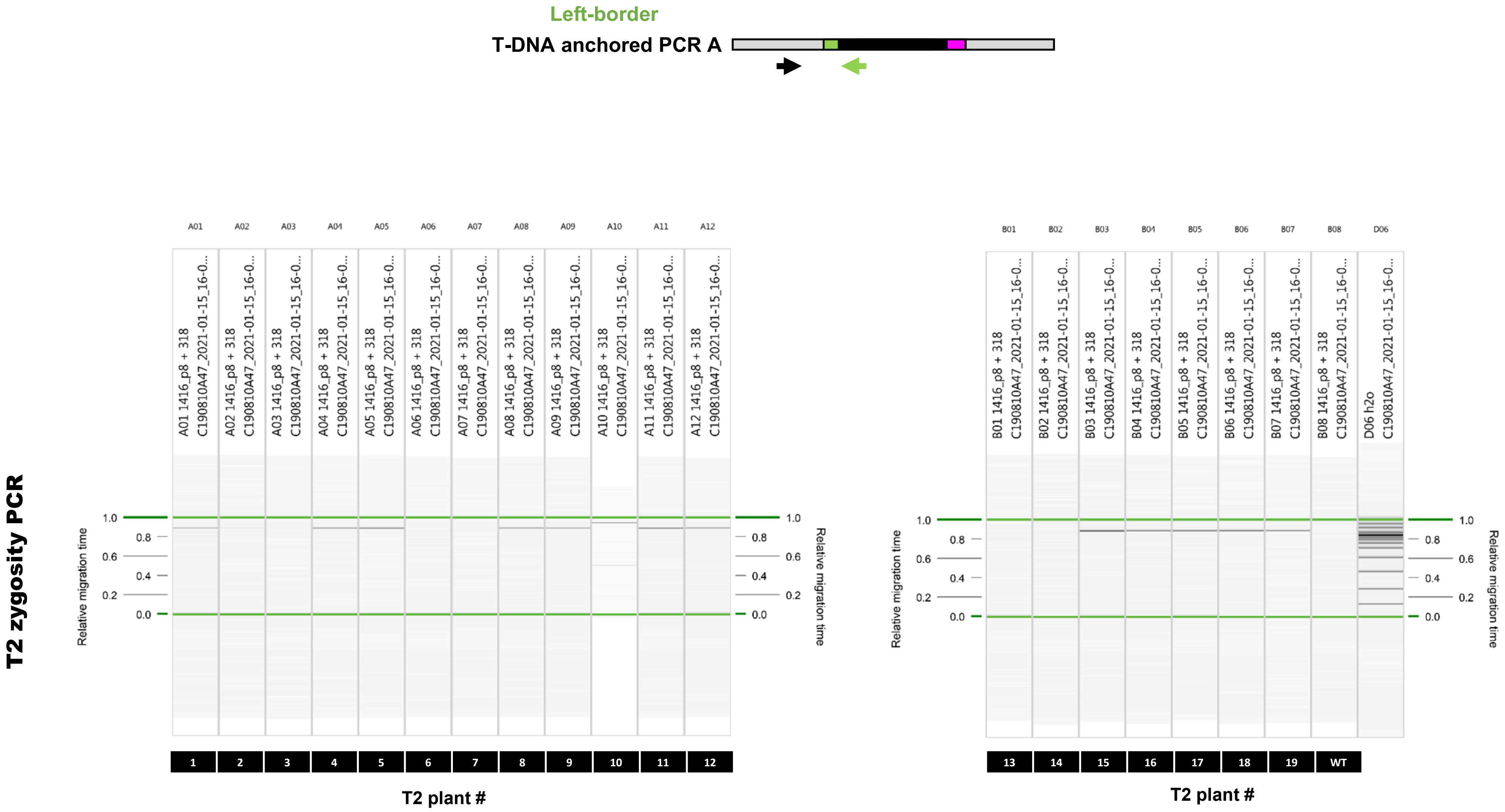

Figure S9

Camelina sativa
Line #1416
OCP1 insertion at Chr15:982591

T-DNA flanking PCR B

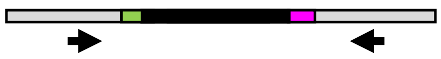

T2 zygosity PCR

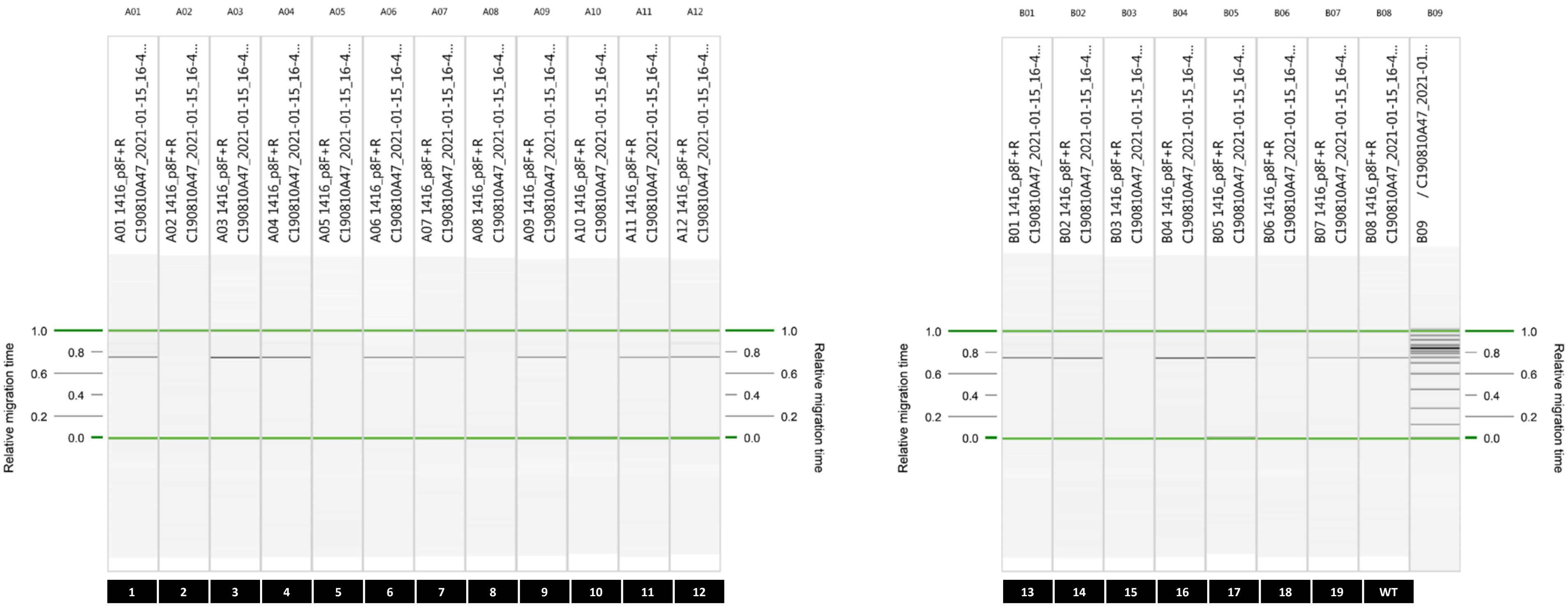

T2 plant # **T2 plant #**
